# HDAC2‐Mediated METTL3 Delactylation Promotes DNA Damage Repair and Chemotherapy Resistance in Triple‐Negative Breast Cancer

**DOI:** 10.1002/advs.202413121

**Published:** 2025-02-14

**Authors:** Xiaoniu He, Yuanpei Li, Jian Li, Yu Li, Sijie Chen, Xia Yan, Zhangrong Xie, Jiangfeng Du, Guoan Chen, Jianbo Song, Qi Mei

**Affiliations:** ^1^ Shanxi Bethune Hospital Shanxi Academy of Medical Sciences Third Hospital of Shanxi Medical University Tongji Shanxi Hospital Taiyuan 030032 China; ^2^ State Key Laboratory of Oncology in South China Guangdong Provincial Clinical Research Center for Cancer Sun Yat‐sen University Cancer Center Guangzhou 510060 China; ^3^ Institute of Molecular Medicine and Experimental Immunology University Clinic of Rheinische Friedrich‐Wilhelms‐University 53127 Bonn Germany; ^4^ Department of Human Cell Biology and Genetics Joint Laboratory of Guangdong‐Hong Kong Universities for Vascular Homeostasis and Diseases School of Medicine Southern University of Science and Technology Shenzhen 518055 China; ^5^ Department of Medical Imaging Shanxi Key Laboratory of Intelligent Imaging and Nanomedicine First Hospital of Shanxi Medical University Taiyuan 030001 China; ^6^ Department of Oncology Tongji Hospital Tongji Medical College Huazhong University of Science and Technology Wuhan 430030 China

**Keywords:** chemotherapy resistance, HDAC2, lactylation, METTL3, TNBC

## Abstract

The current treatment of triple‐negative breast cancer (TNBC) is still primarily based on platinum‐based chemotherapy. However, TNBC cells frequently develop resistance to platinum and experience relapse after drug withdrawal. It is crucial to specifically target and eliminate cisplatin‐tolerant cells after platinum administration. Here, it is reported that upregulated *N* ^6^‐methyladenosine (m^6^A) modification drives the development of resistance in TNBC cells during cisplatin treatment. Mechanistically, histone deacetylase 2 (HDAC2) mediates delactylation of methyltransferase‐like 3 (METTL3), facilitating METTL3 interaction with Wilms’‐tumor‐1‐associated protein and subsequently increasing m^6^A of transcript‐associated DNA damage repair. This ultimately promotes cell survival under cisplatin. Furthermore, pharmacological inhibition of HDAC2 using Tucidinostat can enhance the sensitivity of TNBC cells to cisplatin therapy. This study not only elucidates the biological function of lactylated METTL3 in tumor cells but also highlights its negative regulatory effect on cisplatin resistance. Additionally, it underscores the nonclassical functional mechanism of Tucidinostat as a HDAC inhibitor for improving the efficacy of cisplatin against TNBC.

## Graphical abstract

This study identifies the lactylation modification of methyltransferase‐like 3 (METTL3) in breast tumors and its delactylase histone deacetylase 2 (HDAC2). It elucidates the molecular mechanism by which HDAC2‐mediated METTL3 delactylation promotes triple‐negative breast cancer (TNBC) resistance to cisplatin through upregulation of DNA damage repair pathway. The HDAC inhibitor Tucidinostat is proposed as a potential clinical approach for alleviating TNBC cisplatin resistance.

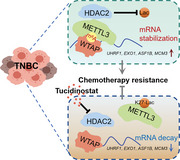

## 1 Introduction

Triple‐negative breast cancer (TNBC) is a heterogeneous breast cancer subtype that does not express estrogen receptor, progesterone receptor, and unamplified epidermal growth factor receptor 2 (HER2), accounting for about 12–17% of diagnosed breast cancer cases.^[^ ^1^ ^]^ Patients with TNBC often do not benefit from endocrine or anti‐HER2 therapy due to lack of expression of these receptors. High early recurrence rates, limited treatment options, and poor prognosis make TNBC the most difficult subtype of breast cancer to treat.^[^ ^2^ ^]^ In the past few decades, taxane/anthracycline chemotherapy has been the primary treatment for TNBC. However, the irreversible cardiotoxicity and drug resistance of anthracyclines have become increasingly apparent.^[^ ^3^ ^]^ Platinum‐based chemotherapy has been the focus of much attention. Platinum compounds belong to cell cycle nonspecific drugs, which mediate the necrosis or apoptosis of tumor cells by inhibiting DNA replication.^[^ ^4^ ^]^ Clinical treatment mainly includes cisplatin, carboplatin, and oxaliplatin. Many patients undergoing cisplatin chemotherapy frequently encounter organ toxicity, which often requires dose reduction or treatment delays.^[^ ^5^ ^]^ Therefore, it is imperative to further investigate the potential of utilizing lower doses of cisplatin or searching for combination drugs to providing a less painful and more effective treatment option.

As a key protein regulating RNA *N* ^6^‐methyladenosine (m^6^A) modification in eukaryotic cells, methyltransferase‐like 3 (METTL3) plays an important biological function in a variety of physiological processes and diseases, especially in the occurrence and development of tumors.^[^ ^6^ ^]^ Inhibition of METTL3 has been proven to be an effective measure in the treatment of various malignant tumors.^[^ ^7^, ^8^ ^]^ However, whether METTL3 is involved in the platinum‐based resistance of TNBC remains unclear. Post‐translational modification (PTM) of protein is an important part of regulating protein function, and SUMO modification of METTL3 inhibits its m^6^A catalytic function.^[^ ^9^ ^]^ METTL3 phosphorylation mediates its deubiquitination and promotes protein stability.^[^ ^10^ ^]^ Our previous work discovers the acetylation modification of METTL3 and demonstrates that acetylation prevents METTL3 nuclear localization and impedes its m^6^A catalytic function of nascent RNA.^[^ ^11^ ^]^ In 2019, lactylation emerged as a novel post‐translational modification of histone lysine residues, mediated by lactate metabolism, and was found to globally regulate gene transcription.^[^ ^12^ ^]^ Subsequent studies have demonstrated that lactylation modification plays critical roles in various pathological processes, including inflammation and cancer progression.^[^ ^13^, ^14^ ^]^ Additionally, enzymes responsible for lactylation, termed writers and erasers, have been progressively identified. Notably, the writers primarily include p300, HBO1, and AARS1/2.^[^ ^12^, ^15^, ^16^, ^17^ ^]^ To date, histone deacetylase 1–3 (HDAC1–3) and Sirtuin 1–3 (SIRT1–3) have been identified as the primary erasers.^[^ ^18^, ^19^, ^20^ ^]^ Recent studies have found that lactylation modification of METTL3 exists in immune cells and is mediated by p300.^[^ ^21^ ^]^ However, the lactylation modification of METTL3 in tumor cells, especially in breast cancer, and its regulatory mechanism and biological function are still unclear.

Here, we observed a significant upregulation of m^6^A modification in cisplatin‐treated TNBC cells during the acquisition of drug resistance, and this dysregulation was attributed to hypolactylated METTL3. Additionally, we identified histone deacetylase 2 (HDAC2), as being involved in mediating delactylation at site K27 of METTL3 in TNBC cells. Subsequently, we demonstrated that HDAC2‐mediated METTL3 delactylation can confer resistance to cisplatin by upregulating the expression of genes associated with DNA damage repair. We demonstrated that the pharmacological inhibition of HDAC2 by Tucidinostat can effectively enhance the efficacy of cisplatin and eradicate TNBC cells both in vitro and in mouse models. Our study will elucidate the biological function of METTL3 lactylation modification in TNBC cells and highlight the nonclassical functional mechanism of Tucidinostat as a HDAC inhibitor for enhancing the efficacy of cisplatin on TNBC.

## 2 Results

### 2.1 RNA m^6^A Modification Is Significantly Upregulated during Cisplatin Tolerance in TNBC Cells

**Figure 1:**
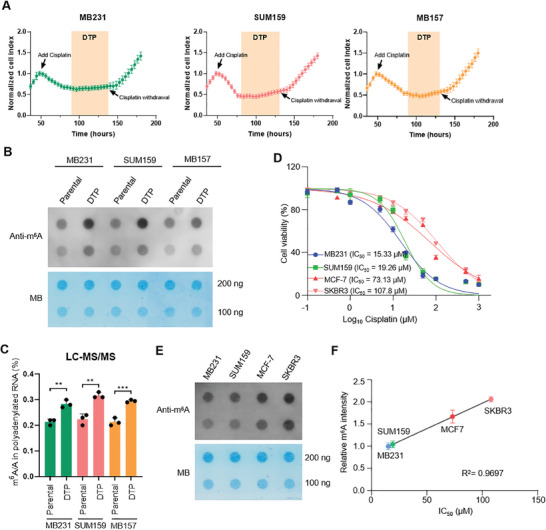
RNA m^6^A modification is significantly upregulated during cisplatin tolerance in TNBC cells. A) MB231 (15 µm cisplatin), SUM159 (20 µm cisplatin), or MB157 (15 µm cisplatin) cells were subjected to ACEA‐xCELLigence real‐time cell proliferation assay for 180 h followed by the indicated drug operation. B) Dot blot of m^6^A in total RNA from paired parental and cisplatin‐tolerant cells using MB231, SUM159, or MB157. MB staining served as RNA loading control in dot blot analysis. C) LC–MS/MS quantification of the m^6^A/A ratio in polyadenylated RNA isolated from the indicated cells. D) Dose–response curves for cisplatin in four breast cancer cell lines as detected by CCK‐8 assay. E) Dot blot of m^6^A in total RNA from indicated cells. MB staining served as RNA loading control in dot blot analysis. F) Correlation analysis comparing m^6^A abundance and cisplatin sensitivity. All data are representative of three independent experiments. Mean ± SD, statistical analysis was performed using two‐tailed Student's *t*‐test (C) or Pearson's coefficient test (F). **p < 0.01, ***p < 0.001.

In order to investigate the mechanism of cisplatin resistance in TNBC, three TNBC cell lines (MB231, SUM159, and MB157) were initially subjected to cisplatin treatment as a form of platinum‐based chemotherapy. Real‐time quantitative analysis of cellular activity revealed that cisplatin could partially eradicate TNBC cells. However, ≈50% of the cells survived from cisplatin treatment and resumed their proliferative state after withdrawal of cisplatin (**Figure** **1**A). This phenomenon suggests that TNBC cells may gradually develop resistance to the cytotoxic effects of cisplatin by entering a drug‐tolerant persister (DTP) state during continuous exposure to the drug, rendering them resistant to its effects.^[^ ^22^ ^]^

Based on the aforementioned phenomenon, we sought to elucidate the molecular mechanisms underlying cisplatin‐induced resistance and subsequent entry into a drug tolerant state in TNBC. In our previous investigations, we have demonstrated that m^6^A modification plays a pivotal role in conferring PARP inhibitor resistance in breast and ovarian cancer by upregulating genes associated with DNA damage repair.^[^ ^23^ ^]^ Given that cisplatin exerts its cytotoxic effects through induction of apoptosis via DNA damage,^[^ ^24^ ^]^ our initial focus was to assess the disparity of m^6^A levels between parental TNBC cells and those residing within the cisplatin‐tolerant state. Utilizing RNA dot blot, we observed a significant upregulation of m^6^A modification in TNBC cells during their transition into the cisplatin‐tolerant state (Figure 1B). The liquid chromatography–tandem mass spectrometry (LC–MS/MS) employed for determining the m^6^A/A ratio of mRNA further validated an elevated global m^6^A modification level in TNBC cells under the cisplatin‐tolerant state (Figure 1C). To delve deeper into understanding the association between intracellular m^6^A modification levels and chemotherapeutic drug sensitivity, we examined the susceptibility of 4 breast cancer cell lines to cisplatin (Figure 1D) while concurrently measuring their corresponding m^6^A modifications (Figure 1E). We observed that SKBR3 and MCF7, two non‐TNBC cell lines, exhibited relatively lower sensitivity to cisplatin compared to the other two TNBC cell lines. Correspondingly, these two cell lines also show higher basal levels of m^6^A (Figure 1F). This indicates a negative correlation between m^6^A abundance and cisplatin sensitivity, thereby suggesting a potential involvement of m^6^A in mediating resistance against cisplatin‐induced cell death.

### 2.2 METTL3 Lactylation Exhibits a Significant Decrease in TNBC Cells upon Cisplatin‐Tolerant State

**Figure 2:**
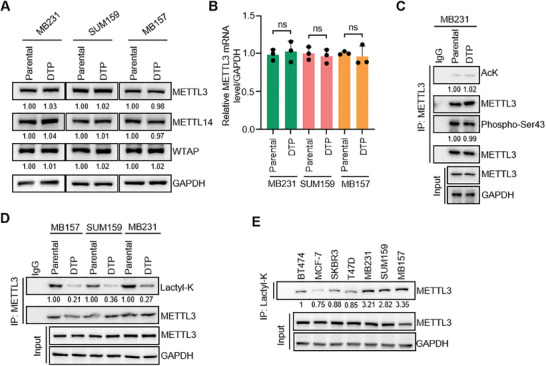
METTL3 lactylation exhibits a significant decrease in TNBC cells upon cisplatin‐tolerant state. A) IB analysis of the indicated proteins in paired parental and cisplatin‐tolerant cells. B) qRT‐PCR quantification of the indicated mRNAs in paired parental and cisplatin‐tolerant cells. C) Lysates of MB231 parental and cisplatin‐tolerant cells were subjected to IP and IB analysis. D) Lysates of paired parental and cisplatin‐tolerant cells were subjected to IP and IB analysis. E) Lysates of multiple breast cancer cell lines were subjected to IP and IB analysis. All data are representative of three independent experiments. Mean ± SD, statistical analysis was performed using two‐tailed Student's *t*‐test (B). ns, no significance.

To investigate the biochemical mechanism underlying the upregulation of m^6^A levels during cisplatin‐tolerant state, we initially compared METTL3 protein and RNA levels among three different TNBC cell lines. Our findings showed no significant alterations in METTL3 protein or RNA levels under cisplatin‐tolerant conditions (**Figure** **2**A,B). Previous studies have demonstrated that low‐expressed METTL3 can effectively suppress tumor growth or metastasis across different cancer types.^[^ ^25^, ^26^, ^27^ ^]^ However, analysis of the TCGA database revealed no significant differences in METTL3 expression among multiple cancers (Figure S1A, Supporting Information). This suggests that PTM of the METTL3 protein may play a crucial role in regulating its function. However, our PTM immunoprecipitation (IP) assay confirmed no significant alterations in acetylation and phosphorylation on METTL3 under cisplatin‐tolerant state (Figure 2C). Although lactylation of METTL3 has been observed in tumor‐infiltrating myeloid cells,^[^ ^21^ ^]^ it remains unclear whether this modification occurs in solid tumor cells, particularly breast cancer cells. We conducted lactylation IP and observed a significant decrease of METTL3 lactylation modification in the cisplatin‐tolerant state (Figure 2D). To elucidate the specificity of METTL3 lactylation reduction in the cisplatin‐tolerant state and the specific association between changes in m^6^A levels and METTL3 lactylation. First, we confirmed that the expression levels of these m^6^A regulatory enzymes (such as METTL14, Wilms’‐tumor 1‐associated protein (WTAP), METTL16, FTO, and ALKBH5) did not significantly differ between cisplatin‐tolerant and parental cells (Figure S1B, Supporting Information). Second, we observed that the lactylation levels of METTL14 and METTL16 were not substantially altered in the cisplatin‐tolerant state, and no lactylation signals were detected on WTAP, FTO, and ALKBH5 (Figure S1B, Supporting Information). This suggests the specificity of METTL3 lactylation change. The presence of basal lactylated METTL3 was detected in various breast cancer types (Figure 2E). In addition, METTL3 has a higher lactylation level in TNBC, which may be related to the higher glycolytic rate and subsequently lactic acid accumulation in TNBC.^[^ ^28^ ^]^ Through the utilization of lactate metabolism inhibitors (Figure S1C, Supporting Information), we discovered a notable reduction in the level of lactylation of METTL3 (Figure S1D, Supporting Information). By contrast, treatment of the cells with lactic acid or lactate resulted in a significant augmentation of the lactylation of METTL3 (Figure S1E, Supporting Information). In summary, METTL3 is lactylated in breast cancer, and lactylation level of METTL3 is significantly reduced during TNBC cells entering a cisplatin‐tolerant state.

### 2.3 METTL3 Is Directly Delactylated at K27 by HDAC2 in TNBC

**Figure 3:**
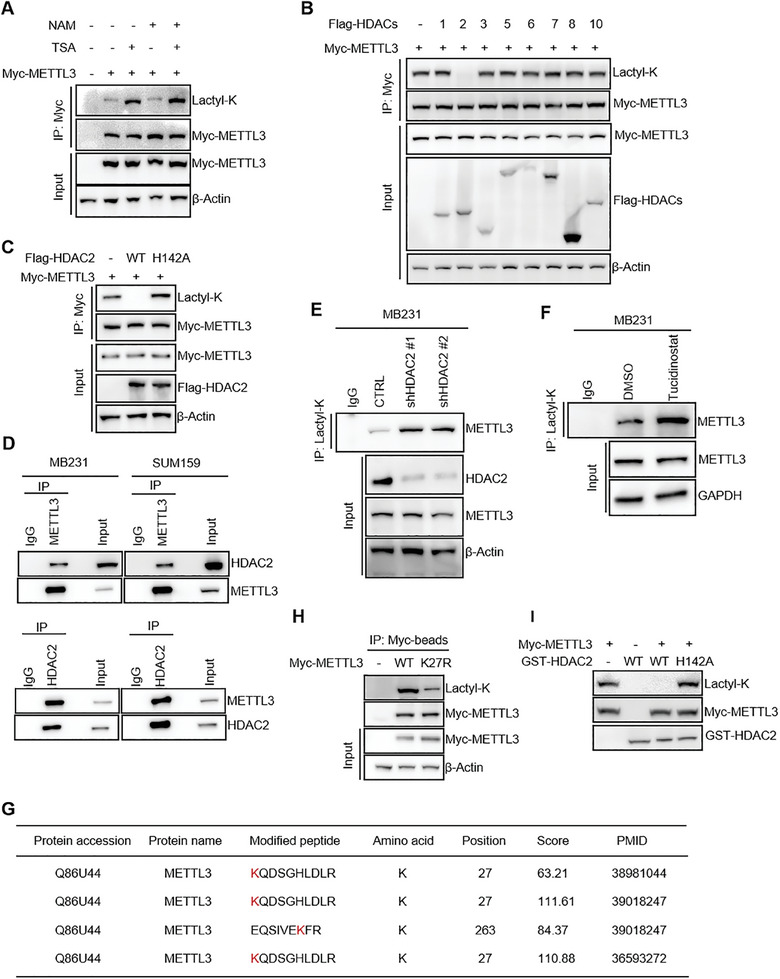
METTL3 is directly delactylated at K27 by HDAC2 in TNBC. A) HEK293T cells transfected with the indicated constructs were treated with TSA (1 µm) or/and NAM (5 mm) for 6 h. Lysates were subjected to IP and IB analysis. B) Lysates of HEK293T cells transfected with the indicated constructs were subjected to IP and IB analysis. C) Lysates of HEK293T cells transfected with the indicated constructs were subjected to IP and IB analysis. D) Co‐IP of HDAC2 with METTL3 in MB231 or SUM159 cells. The immunoprecipitated materials by the indicated antibodies were analyzed by western blotting. E) IP and IB analysis of MB231 cells infected with the indicated lentiviruses. F) IP and IB analysis of MB231 cells treated with HDAC2 inhibitor Tucidinostat (1 µm) for 72 h. G) The table provides an overview of the lactylation sites of METTL3 as depicted in the three proteomic datasets. H) Lysates of HEK293T cells transfected with the indicated constructs were subjected to IP and IB analysis. I) Incubation of the indicated recombinant HDAC2 proteins with lactylated Myc‐METTL3 proteins, followed by IB analysis of the lactylation level of METTL3 with pan‐lactyl lysine (Kla) antibody. All data are representative of three independent experiments.

To investigate the upstream regulatory molecules responsible for the reduction in METTL3 lactylation during cisplatin treatment. Initially, we aim to understand whether the reduction in METTL3 lactylation during cisplatin tolerance is regulated by its lactyltransferase p300. We utilized shRNA targeting p300 to confirm that the knockdown of p300 leads to a reduction in the lactylation levels of H3K18la, a known substrate of p300 lactylation, as well as METTL3, in MB231 cells (Figure S2A, Supporting Information). We observed that p300 expression levels or lactyltransferase activity (characterized by H3K18la) in cisplatin‐tolerant cells did not differ from those in parental cells (Figure S2B, Supporting Information). Intracellular lactate concentrations play a crucial role in the initiation of lactylation modifications. However, no significant differences in lactate levels were observed between the parental and cisplatin‐tolerant cells (Figure S2C, Supporting Information). It suggests that the decrease in METTL3 lactylation levels in cisplatin‐tolerant breast cancer cells is not due to reduced “installation” but rather increased “erasure.” And then it was hypothesized that direct regulation of METTL3 by the delactylase may occur. Therefore, this study employed HDAC family pan‐inhibitor Trichostatin A (TSA) and SIRT family pan‐inhibitor Nicotinamide (NAM) treatments on cells.^[^ ^20^ ^]^ Surprisingly, the lactylation modification of METTL3 was increased only under TSA‐treated conditions, suggesting that the delactylase of METTL3 may be derived from HDAC family proteins (**Figure** **3**A). By constructing and transfecting a HDAC protein expression library, the lactylation IP experiment showed that HDAC2 significantly reduced the level of lactylation on METTL3 (Figure 3B). When the catalytic site of HDAC2 was mutated (H142A), the delactylation of METTL3 was abrogated (Figure 3C). Co‐IP experiments revealed an interaction between endogenous HDAC2 and METTL3 in MB231 and SUM159 cells (Figure 3D). This is also the case in other TNBC cell lines such as MB157 and Hs578T (Figure S2D, Supporting Information). After interfering the expression of endogenous HDAC2 by shRNA, lactylation on METTL3 was significantly increased (Figure 3E and Figure S2E, Supporting Information). Additionally, treatment with Tucidinostat, a HDAC inhibitor (HDACi), also led to a significant increase of lactylation on METTL3 (Figure 3F and Figure S2F, Supporting Information).

Next, we aim to further investigate the key site of METTL3 delactylation mediated by HDAC2. Previously, a total of 110 clinical hepatocellular carcinoma samples were collected for lactylation modification mass spectrometry analysis,^[^ ^29^ ^]^ resulting in the identification of 9140 proteins with lactylation modifications and a cumulative count of 9275 lactylation modification sites. Among these sites, it was observed that the K27 site of METTL3 underwent lactylation. Furthermore, clinical samples from six cases of lung cancer and their corresponding adjacent cancers were collected for lactylation proteomics analysis,^[^ ^30^ ^]^ leading to the identification of 806 proteins with a combined total count of 2193 lactylated modification sites. In terms of gastrointestinal cancer analysis,^[^ ^31^ ^]^ a set comprising 40 cases along with their corresponding adjacent tissues was utilized to identify a total number of 3156 proteins out of an initial pool consisting initially identified as having a cumulative count reaching up to 11 698 lactylated sites (Figure 3G). K27 of METTL3 turns out to be a conserved residue among different species (Figure S2G, Supporting Information). The lactylation‐modified omics data collectively support the notion that METTL3 undergoes lactylation modification in tumors, with lysine 27 being identified as a potential site. To validate this, we generated a K27 mutant of METTL3 and performed an IP assay in 293T cells. Wild‐type METTL3 can detect a significant lactylation signal with pan‐lactylated lysine antibodies, but lactylation is almost undetectable when K is mutated to R (Figure 3H).

Following this, we isolated lactylated METTL3 from the cells and performed an in vitro incubation with recombinant HDAC2 protein. During this process, wild‐type HDAC2, but not HDAC2–H142A, directly catalyzed the removal of the lactoyl group from lactylated METTL3 (Figure 3I). However, when subjected to incubation of METTL3–K27R and HDAC2 recombinant proteins, the delactylation reaction failed to further enhancement compared to incubation of wild‐type METTL3 and HDAC2 (Figure S2H, Supporting Information). Our findings demonstrate that HDAC2 is responsible for delactylating METTL3 at the K27 site.

### 2.4 METTL3 K27 Delactylation by HDAC2 Promotes Integrity of the methyltransferase complex (MTC) and m^6^A Modification upon Cisplatin Treatment

**Figure 4:**
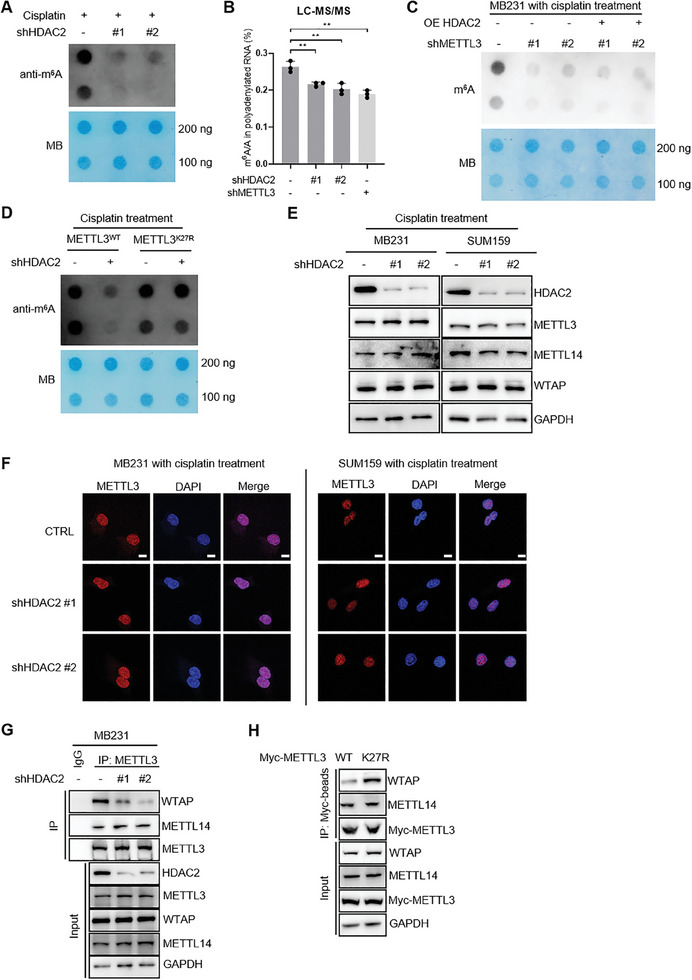
METTL3 K27 delactylation by HDAC2 promotes integrity of the MTC and m^6^A modification upon cisplatin treatment. A) Dot blot of m^6^A in total RNA in MB231 cells after shRNA knockdown of HDAC2 under cisplatin treatment (1 µm) for 24 h. B) LC–MS/MS quantification of the m^6^A/A ratio in polyadenylated RNA isolated from the indicated MB231 cells combined cisplatin treatment (1 µm) for 24 h. C) Dot blot of m^6^A in total RNA from indicated cells combined cisplatin treatment (1 µm) for 24 h. D) Dot blot of m^6^A in total RNA after shHDAC2 knockdown in METTL3 reconstituting MB231 cells combined cisplatin treatment (1 µm) for 24 h. E) IB analysis of the indicated proteins in MB231 and SUM159 cells infected with the indicated lentiviruses combined cisplatin treatment (1 µm) for 24 h. F) Representative immunofluorescence for METTL3 (red) and DAPI (blue, cell nuclei) in the indicated cell lines infected with the indicated lentiviruses combined cisplatin treatment (1 µm) for 24 h. Scale bars, 10 µm. G) IP and IB analysis of MB231 cells infected with the indicated lentiviruses combined cisplatin treatment (1 µm) for 24 h. H) IP and IB analysis of METTL3 reconstituting MB231 cells combined cisplatin treatment (1 µm) for 24 h. All data are representative of three independent experiments. Mean ± SD of *n* = 3 independent experiments by two‐tailed Student's *t*‐test (B). **p < 0.01.

To investigate the impact of METTL3 delactylation on its protein function and the underlying molecular mechanism, we initially employed RNA dot blot for low‐dose cisplatin treatment after knocking down HDAC2. This manipulation led to a significant reduction in intracellular m^6^A modification levels (**Figure** **4**A). The determination of m^6^A/A ratio of mRNA using LC–MS/MS further confirmed that global m^6^A modification level was relatively diminished (Figure 4B). Moreover, in the absence of METTL3, the overexpression of HDAC2 did not result in an increase in global m^6^A levels (Figure 4C and Figure S3A, Supporting Information). No additional decrease in m^6^A levels was observed after HDAC2 knockdown in METTL3‐deficient MB231 cells under cisplatin treatment (Figure S3B,C, Supporting Information), indicating that HDAC2 regulates attenuation of intracellular m^6^A modifications via METTL3. To evaluate the direct contribution of K27 lactylation for decreased m^6^A, METTL3‐depleted MB231 cells were reconstituted with either METTL3^WT^ or METTL3^K27R^ (nonlactylation mimetic) constructs. The m^6^A level of METTL3^WT^ cells could be significantly reduced by HDAC2 knockdown under cisplatin treatment, whereas no significant reduction was observed in METTL3^K27R^ cells (Figure 4D and Figure S3D Supporting Information).

To comprehend how HDAC2‐mediated METTL3 delactylation regulates m^6^A modification, western blotting and quantitative polymerase chain reaction (qPCR) analysis demonstrated no significant alterations in the protein and RNA levels of core components within the MTC following HDAC2 knockdown upon cisplatin treatment in MB231 and SUM159 cells (Figure 4E and Figure S3E,F, Supporting Information). The nuclear–cytoplasmic distribution of METTL3 remained unchanged after knocking down HDAC2 in MB231 and SUM159 during cisplatin treatment (Figure 4F and Figure S3G, Supporting Information). Intriguingly, a notable decrease in the interaction between METTL3 and WTAP was observed after HDAC2 knockdown under cisplatin treatment both in MB231 and SUM159 cells (Figure 4G and Figure S3H, Supporting Information). WTAP serves as an essential constituent of MTC responsible for guiding MTC toward mRNA targets and supporting its catalytic activity.^[^ ^32^ ^]^ Lactylation of METTL3, induced by HDAC2 knockdown, may compromise the integrity of the MTC and impair RNA m^6^A modification by disrupting its interaction with WTAP. To further investigate whether interaction of METTL3 with WTAP is regulated by HDAC2 through delactylation of the METTL3 K27 site, the interaction between METTL3^K27R^ and WTAP was significantly stronger than that of METTL3^WT^ in reconstituting MB231 cells during cisplatin (Figure 4H). In conclusion, during cisplatin stress, HDAC2 promotes the interaction between METTL3 and WTAP by delactylating METTL3 at K27, thereby augmenting intracellular m^6^A levels.

### 2.5 The Delactylation of METTL3 by HDAC2 Contributes to the Development of Cisplatin Resistance in TNBC

**Figure 5:**
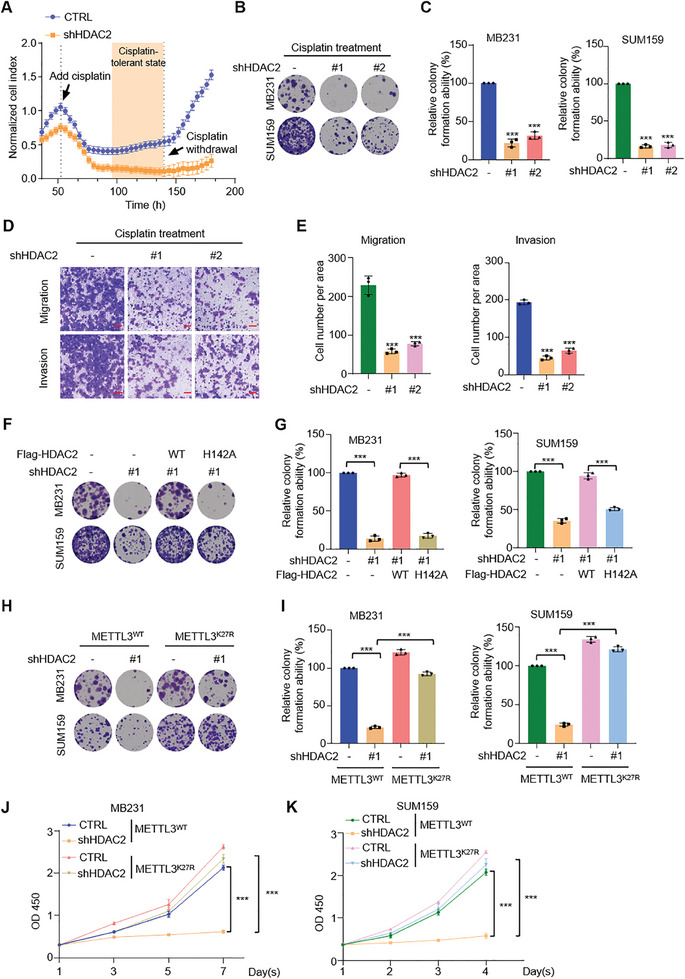
Delactylation of METTL3 by HDAC2 contributes to the development of cisplatin resistance in TNBC. A) MB231 cells infected with the indicated lentiviruses were subjected to ACEA‐xCELLigence real‐time cell proliferation assay for 180 h followed by the indicated drug operation (15 µm cisplatin). B,C) Clonogenic assay and quantification of the indicated MB231 and SUM159 cells subjected to the indicated lentiviruses combined cisplatin treatment (0.5 µm) for 14 days. D,E) Migration/invasion assays and quantification were performed in the indicated MB231 cells infected with indicated lentiviruses combined cisplatin treatment (0.5 µm). Scale bars, 50 µm. F,G) Clonogenic assay and quantification of the indicated MB231 and SUM159 cells subjected to the indicated lentiviruses combined cisplatin treatment (0.5 µm) for 14 days. H,I) Clonogenic assay and quantification of the indicated METTL3 reconstituting MB231 and SUM159 cells subjected to the indicated lentiviruses combined cisplatin treatment (0.5 µm) for 14 days. J,K) Cell proliferation assays for METTL3 reconstituting MB231 and SUM159 cells infected with indicated lentiviruses combined cisplatin treatment (1 µm). All data are representative of three independent experiments. Mean ± SD, statistical analysis was performed using two‐tailed Student's *t*‐test (C, E, G, I–K). ***p < 0.001.

After analyzing the TCGA database, we discovered that HDAC2 was highly expressed in more than two‐thirds of tumors, including breast cancer (Figure S4A,B, Supporting Information). Furthermore, high expression of HDAC2 in breast cancer significantly correlated with a negative impact on overall survival (Figure S4C, Supporting Information), indicating the crucial role of HDAC2 as a potential target for treating breast cancer. To further investigate the involvement of HDAC2 in cisplatin chemotherapy for TNBC cells, we initially demonstrated that fewer cells after lacking HDAC2 entered the DTP state during cisplatin treatment through real‐time cell quantification (**Figure** **5**A). The clonogenesis assay revealed that TNBC cells with depleted HDAC2 exhibited significantly reduced clonogenicity when exposed to cisplatin (Figure 5B,C). The Cell Counting Kit‐8 (CCK‐8) experiment further substantiated the diminishing efficacy of its proliferation capability (Figure S4D,E, Supporting Information). Moreover, Transwell experiments demonstrated that knockdown of HDAC2 effectively inhibited migration and invasion abilities of breast cancer cells during cisplatin treatment (Figure 5D,E). These findings highlight the critical role played by HDAC2 in mediating cisplatin resistance in TNBC.

In order to further validate the aforementioned conclusions, we conducted experiments by replacing shRNA‐resistant HDAC2 wild‐type and H142A after HDAC2 knockdown. First, we assessed the knockdown and rescue effects of HDAC2 using western blotting analysis (Figure S4F, Supporting Information). Consistent with above findings, knockdown of HDAC2 significantly suppressed clonogenesis and cell proliferation in TNBC cells during cisplatin treatment. Moreover, supplementation of HDAC2^WT^ effectively restored the phenotypic changes caused by HDAC2 knockdown. However, replacement of catalytic mutants (H142A) of HDAC2 failed to reverse the phenotypic alterations induced by HDAC2 knockdown (Figure 5F,G and Figure S4G,H, Supporting Information). These results confirm that through its acyltransferase activity, HDAC2 can confer resistance to cisplatin treatment in TNBC cells.

To investigate whether HDAC2 regulates cisplatin resistance in TNBC cells by delactylating METTL3 K27, the METTL3^WT^ and METTL3^K27R^ reconstituting MB231 cells treated with cisplatin were utilized for colony formation and cell proliferation assay. The results of both MB231 and SUM159 cell lines demonstrated that lactylation at K27 site of METTL3 was essential for sensitizing TNBC cells to cisplatin (Figure 5H–K). In conclusion, delactylation of METTL3 K27 site mediated by HDAC2 plays a crucial role in promoting growth and metastasis of TNBC cells as well as conferring drug tolerance during cisplatin chemotherapy.

### 2.6 The Delactylation of METTL3 by HDAC2 Enhances the Expression of Gene‐Associated DNA Damage Repair in TNBC during Cisplatin Treatment

**Figure 6:**
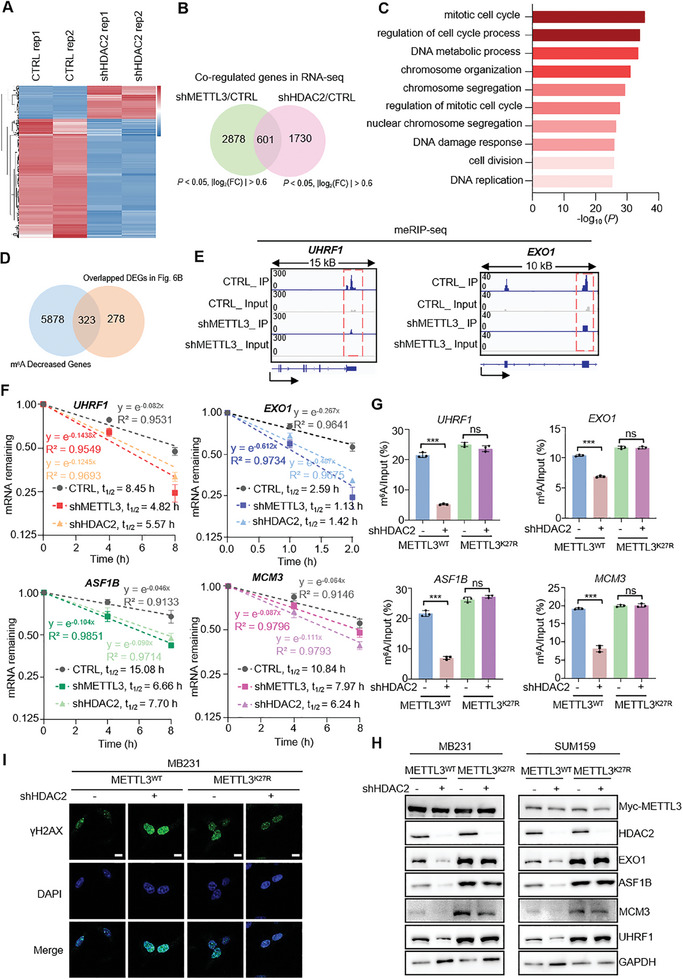
Delactylation of METTL3 by HDAC2 enhances the expression of genes associated DNA damage repair in TNBC during cisplatin treatment. A) Heatmap of differential expressed genes (FDR < 0.05) among control or HDAC2 depleted MB231 cells. B) Venn diagram showing overlaps of HDAC2 regulated and METTL3 regulated genes. RNA‐seq data of METTL3‐deficient and Control MB231 cells were obtained from GEO database (GSE183017). C) Gene ontology functional enrichment analysis of overlapped genes in (B). D) Venn diagram showing m^6^A decreased genes from meRIP‐seq of METTL3‐deficient and Control MB231 cells overlapped with 601 genes in (B). meRIP‐seq data of METTL3‐deficient and Control MB231 cells were obtained from GEO database (GSE183017). E) Genomic visualization of the meRIP‐seq normalized signal in METTL3‐dificient MB231 cells for the METTL3‐dependent m^6^A substrates UHRF1, EXO1. Blue, meRIP; gray, input. F) Reducing UHRF1, EXO1, ASF1B, and MCM3 mRNA half‐life by silencing METTL3 or HDAC2 in MB231 cells. G) m^6^A levels in UHRF1, EXO1, ASF1B, and MCM3 transcripts determined by gene‐specific m^6^ARIP–qPCR assays in METTL3 reconstituting MB231 cells infected shHDAC2 or control lentiviruses. H) IB analysis of the indicated proteins in METTL3 reconstituting MB231 and SUM159 cells infected with indicated lentiviruses. I) Representative images of γH2AX foci in METTL3 reconstituting MB231 infected with indicated lentiviruses upon 1 µm cisplatin treatment for 24 h. Scale bars, 10 µm. Values are mean ± SD of *n* = 3 independent experiments (F–I). Statistical analysis was performed using two‐tailed Student's *t*‐test (G). ***p < 0.001. ns, no significance.

To further investigate the downstream signaling pathway regulated by METTL3 delactylation to tolerate cytotoxicity of cisplatin, we collected total RNA from MB231 cells with silencing HDAC2 and performed high‐throughput RNA sequencing (**Figure** **6**A). Differential expressed genes (HDAC2 knockdown compared to the control group, |log_2_(FC)| > 1 and *p* < 0.05) were subjected to Gene ontology (GO) analysis (Figure S5A, Supporting Information). Downstream target genes regulated by METTL3 in our previous RNA‐seq of MB231 with METTL3 knockdown (GSE183017) were overlapped with those affected by HDAC2, a total of 601 coregulated genes being identified at the mRNA level (Figure 6B). GO analysis of overlapped genes revealed an enrichment in cell growth and DNA damage response (Figure 6C), suggesting that HDAC2 and METTL3 may play a common role in cancer survival. Decreased m^6^A peaks from meRIP‐seq data (GSE183017) of METTL3 knockdown were used to overlap with the 601 downstream target genes, resulting in the identification of a total of 323 downstream target genes mediated by m^6^A (Figure 6D). Among these, UHRF1, EXO1, ASF1B, and MCM3 were found to be downstream target genes related to DNA damage repair (Figure S5B, Supporting Information).^[^ ^33^, ^34^, ^35^, ^36^ ^]^ The m^6^A signals of these representative transcripts significantly decreased after silencing METTL3 (Figure 6E and Figure S5C Supporting Information). MeRIP–qPCR analysis was utilized to validate the significant decreases in m^6^A deposition of the selected transcripts in cells depleted of METTL3 or HDAC2 in MB231 and SUM159 cells (Figure S5D,E, Supporting Information). qRT‐PCR results verified downregulation of these target transcripts upon HDAC2 or METTL3 depletion (Figure S5F,G, Supporting Information). m^6^A has been reported to increase mRNA stability and/or translation.^[^ ^37^ ^]^ mRNA half‐life measurement assay suggested that both the RNA stability of UHRF1, EXO1, ASF1B, and MCM3 decreased significantly upon HDAC2 knockdown in MB231 and SUM159 cells (Figure 6F and Figure S5H Supporting Information). To investigate the contribution of METTL3 delactylation by HDAC2 in HDAC2‐mediated m^6^A enrichment change, we analyzed m^6^A levels of the selected 4 genes in METTL3 reconstituting cells (Figure 6G). Validation of their mRNA levels revealed that, compared to METTL3^WT^ reconstitution, METTL3^K27R^ conferred resistance to HDAC2 depletion‐induced alteration of their transcripts expression (Figure S5I,J, Supporting Information). Also, for these genes, changes in their protein expression patterns were quite consistent with those in RNA levels (Figure 6H). Depletion of HDAC2 under cisplatin treatment induces DNA damage in METTL3^WT^ cells, but not in METTL3^K27R^ cells (Figure 6I and Figure S5K, Supporting Information). Additionally, an analysis of the TCGA database revealed a significant positive correlation between HDAC2 and the four target genes in breast cancer tumor tissues (*n* = 1085) (Figure S5L, Supporting Information). To investigate whether HDAC2‐mediated METTL3 delactylation regulates DNA damage repair through UHRF1, EXO1, ASF1B, and MCM3. As shown in Figure S6 (Supporting Information), exogenous overexpression of UHRF1, EXO1, ASF1B, or MCM3 can significantly yet partially reversed DNA damage and colony conformation triggered by HDAC2 silencing. These results highlight that HDAC2‐mediated delactylation of METTL3 at K27 site promotes DNA damage repair and confers cisplatin resistance in TNBC.

### 2.7 The Pharmacological Inhibition of HDAC2 Enhances the Sensitivity of TNBC Cells to Cisplatin through Maintaining METTL3 Lactylation

We subsequently assessed the potential anti‐TNBC effects of combining HDAC inhibitor with cisplatin. Tucidinostat, an approved HDAC inhibitor for hematological tumors,^[^ ^38^ ^]^ may also be utilized in combination with aromatase inhibitors for hormone receptor‐positive, human epidermal growth factor receptor‐2 negative, postmenopausal patients with locally advanced or metastatic breast cancer who have experienced relapse or progression on endocrine therapy.^[^ ^39^, ^40^, ^41^ ^]^ However, there is currently insufficient conclusive evidence supporting the utilization of Tucidinostat in TNBC. Our previous studies have demonstrated that HDAC2 can regulate the expression of DNA‐damage‐repair‐related genes such as ASF1B and EXO1 through METTL3, thereby promoting resistance to cisplatin in TNBC. Here, we investigate whether Tucidinostat can exert antitumor effects in combination with cisplatin therapy through this mechanism.

**Figure 7:**
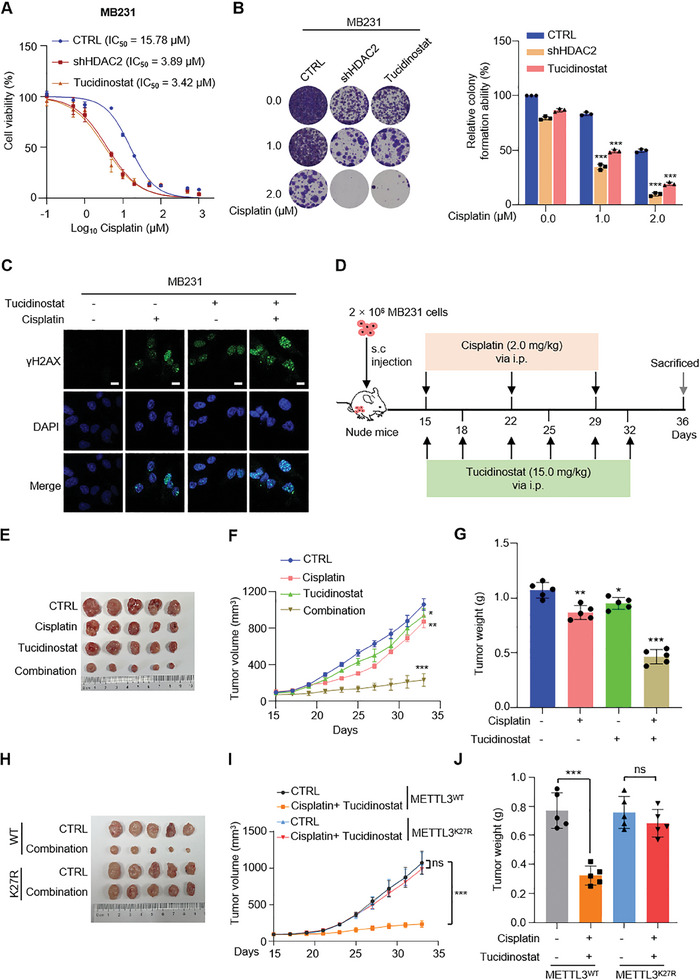
The pharmacological inhibition of HDAC2 enhances the sensitivity of TNBC cells to cisplatin through maintaining METTL3 lactylation. A) Cisplatin dose–response curves for MB231 cells infected with HDAC2 shRNA or treated with 0.5 µm Tucidinostat as detected by CCK‐8 assay. B) Cisplatin dose–response for clonogenic assay and quantification of MB231 subjected to HDAC2 shRNA or 0.5 µm Tucidinostat administration for 14 days. C) Representative images of γH2AX foci in MB231 subjected to 0.5 µm Tucidinostat administration or/and 1 µm cisplatin treatment for 24 h. Scale bars, 10 µm. D) Experimental scheme of drug administration of indicated MB231 xenografted mouse model. E–G) MB231 cells were subcutaneously injected into BALB/c nude mice (*n* = 5). Tumor images were shown in (E); tumor volumes were calculated as described in (F); tumor weights were measured in (G). H–J) METTL3 reconstituting MB231 cells were subcutaneously injected into BALB/c nude mice (*n* = 5). Tumor images were shown in (H); tumor volumes were calculated as described in (I); tumor weights were measured in (J). Data are representative of three independent experiments (A–C). Mean ± SD, statistical analysis was performed using two‐tailed Student's *t*‐test (B, F, G, I, J). *p < 0.05, **p < 0.01, ***p < 0.001. ns, no significance.

By determining the IC50 by CCK‐8 assay, Tucidinostat significantly improved the killing ability of cisplatin on TNBC cells (**Figures** **7**A and S7A, Supporting Information). Tucidinostat combined with cisplatin can further decrease colony formation ability of TNBC cells (Figure 7B and Figure S7B, Supporting Information). The combination of Tucidinostat significantly augmented DNA damage in MB231 and SUM159 cells compared to cisplatin alone (Figures 7C and S7C, Supporting Information). When the K27 site of METTL3 was mutated (which prevents lactylation of METTL3) in MB231 and SUM159 cells, the combination of Tucidinostat and cisplatin could not effectively kill the cells (Figure S7D,E, Supporting Information).

To assess the efficacy of Tucidinostat in combination with cisplatin in vivo, we established a nude mice model with transplanted MB231 cells. The mice were then treated with intraperitoneal injections of low‐dose cisplatin (2 mg kg^−1^) and/or Tucidinostat (15 mg kg^−1^) (Figure 7D). Following one month of combined administration, we observed that cisplatin alone slowed tumor growth, while the combination of Tucidinostat significantly inhibited TNBC tumor cell growth (Figure 7E–G). Subsequently, we collected tumor tissues from the drug‐treated mice and performed protein extraction. Remarkably, the expression levels of DNA‐damage‐repair‐related proteins were significantly reduced in the group treated with cisplatin‐combined Tucidinostat (Figure S6F, Supporting Information). And then, we engineered METTL3 wild‐type and K27R mutant reconstituting MB231 cells to subcutaneous tumor formation in mice. Upon treatment with a combination of cisplatin and Tucidinostat, tumors harboring wild‐type METTL3 were significantly suppressed, whereas those with the K27R mutation, which prevents lactylation of METTL3, showed no significant inhibition (Figure 7H–J). These findings indicate that the efficacy of Tucidinostat in combination with cisplatin on breast cancer tumors is dependent on lactylation modification at the K27 site. Tucidinostat could represent a promising strategy for minimizing the therapeutic dosage of cisplatin in TNBC, warranting further investigation in clinical settings.

Furthermore, we aim to investigate whether the combination of Tucidinostat and cisplatin can exert a therapeutic effect on TNBC that has already developed drug resistance. We induced cisplatin‐resistant MB231 cells (MB231‐R) which had an IC50 approximately fivefold higher at 77.39 µm than that of parental cells at 15.10 µm (Figure S8A, Supporting Information). Similar to cisplatin‐tolerant cells, the lactylation level of METTL3 in the resistant cells was found to be lower compared to that in the parental cells (Figure S8B, Supporting Information). Moreover, m^6^A levels were higher in resistant cells (Figure S8C, Supporting Information). Colony formation assays demonstrated that the combination of Tucidinostat and cisplatin significantly reduced the viability of MB231‐R cells (Figure S8D,E, Supporting Information). We evaluated the efficacy of the in vivo combination of Tucidinostat and cisplatin in a mouse xenograft tumor model using MB231‐R cells (Figure S8F, Supporting Information). The combination of Tucidinostat and cisplatin significantly inhibited the growth of MB231‐R tumors (Figure S8G–I, Supporting Information). These results suggest that even if no intervention is made in the early stage of cisplatin treatment, breast cancer cells that have developed cisplatin resistance remain sensitive to the combination of cisplatin and Tucidinostat.

## 3 Discussion

Cisplatin is considered a “paragon of high efficacy and toxicity,” exhibiting broad indications and commendable effectiveness. However, its potent pharmacological effects have led to increasingly intense adverse reactions.^[^ ^42^ ^]^ Notably, cisplatin not only interacts with cancer cell DNA but also affects the DNA of normally proliferating cells. Nevertheless, due to platinum's accumulation as a heavy metal within the body, cisplatin can disrupt normal physiological activities and even inflict damage on other human tissues. Cardiotoxicity, nephrotoxicity, and ototoxicity represent common manifestations thereof.^[^ ^43^ ^]^ While increasing cisplatin concentration may enhance cancer cell apoptosis rates, it concurrently exacerbates harm inflicted upon the human body. Consequently, identifying strategies to reduce clinical cisplatin concentrations becomes paramount for alleviating chemotherapy‐related distress experienced by cancer patients. In our study, we identified the underlying mechanism by which cisplatin, as a stress, even at low dose, induces METTL3 delactylation and MTC integrity in TNBC cells, thereby enhancing DNA damage repair capacity. By combining subtherapeutic doses of cisplatin with Tucidinostat, we were able to effectively control the growth of TNBC cells both in vitro and in animal models. This finding presents a potential strategy for mitigating adverse reactions and alleviating patient discomfort.

Acquired drug resistance hinders the attainment of stable and complete efficacy in cancer treatment. Recent insights into drug‐tolerant persister cells suggest that nonmutational resistance mechanisms are crucial for the long‐term survival of residual cells following chemotherapy or targeted therapy. Unlike the completely resting phenotype of dormant tumor cells, a subset of DTP cells exposed to treatment is ready to enter the cell cycle, thus promoting disease recurrence after treatment ends.^[^ ^22^ ^]^ Targeting drug tolerant cells thus presents a therapeutic opportunity to prevent recurrence. Previous groundbreaking research has revealed that tolerant cells become reliant on GPX4, and disruption of GPX4 function induces ferroptosis in DTP cells, effectively preventing tumor recurrence in mice.^[^ ^44^ ^]^ Despite the availability of existing GPX4 inhibitors such as RSL3 and ML210 as valuable tool compounds in cell culture settings,^[^ ^45^, ^46^ ^]^ their limited pharmacokinetic properties have impeded their application in vivo. Therefore, it is imperative to explore novel therapeutic vulnerabilities targeting these drug‐tolerant cells. Based on our research experience of RNA epigenetic modification, we initiated an investigation into the m^6^A modification between TNBC cell parents and cisplatin‐tolerant cell lines. Our findings revealed a significant elevation in the m^6^A modification level within the latter, indicating their acquisition of m^6^A dependence as a defense mechanism against cisplatin‐induced DNA damage. Despite numerous studies^[^ ^7^, ^47^, ^48^, ^49^ ^]^ and clinical trials (NCT05584111)^[^ ^50^ ^]^ focusing on m^6^A inhibitors, their application has not yet received clinical approval. Nevertheless, it is noteworthy that we have identified the upstream regulatory mechanism of m^6^A in cisplatin‐tolerant cells, wherein HDAC2 leads to delactylation of METTL3 for regulating the level of m^6^A. HDAC2 inhibition, in combination with cisplatin treatment, augments DNA damage and significantly suppresses TNBC growth. The small molecule inhibitor of HDAC2, Tucidinostat, has demonstrated clinical efficacy in the management of leukemia.^[^ ^38^ ^]^ Henceforth, the coadministration of Tucidinostat and cisplatin holds potential as a therapeutic approach for TNBC.

HDAC2 was initially believed to function as a histone deacetylase, forming multiprotein complexes responsible for the deacetylation of lysine residues at the N‐terminal regions of core histones.^[^ ^51^ ^]^ The removal of histone acetylation further impacts the accessibility of chromatin and consequently inhibits transcription of specific genes.^[^ ^52^ ^]^ It also interacts with various proteins, including YY1, a mammalian zinc‐finger transcription factor, to form repressor complexes, thereby playing a crucial role in transcriptional regulation.^[^ ^53^ ^]^ Moreover, HDAC2 has the ability to deacetylate nonhistone proteins. Deletion or pharmacological inhibition of the HDAC2 gene prevents PD‐L1 nuclear translocation by maintaining acetylation modification on Lys263, thus abolishing PD‐L1 function as a transcription factor involved in regulating other immune checkpoints.^[^ ^54^ ^]^ Our study revealed a novel regulatory mechanism of gene expression by HDAC2, involving its modulation of RNA epigenetic modifications through delactylation. This process impacts RNA stability and ultimately governs gene expression. The delactylation activity of HDAC2 on METTL3 enhances the its m^6^A catalytic activity, resulting in m^6^A modification on transcripts associated with DNA damage repair, thereby improving their RNA stability. Consequently, the upregulation of these genes contributes to cisplatin resistance of TNBC cells.

Recent studies have demonstrated the involvement of lactylation in the regulation of DNA damage repair. Lactylation of the DNA repair protein XRCC1 by the Warburg effect, ultimately facilitates DNA repair and tumor progression.^[^ ^55^ ^]^ Lactylation at site K673 of MRE11 enhances binding with DNA, thereby augmenting cleavage of DNA ends and homologous recombination repair.^[^ ^56^ ^]^ Additionally, lactylated NBS1 protein at site K388 exhibits enhanced recruitment and assembly capabilities for MRN complex, promoting recruitment of HRR proteins at sites of DNA double‐strand breaks and consequently leading to chemoradiation resistance.^[^ ^57^ ^]^ Notably, lactylation plays a pivotal role in mediating therapeutic resistance exhibited by various DNA damage repair proteins. Our study has revealed that lactylation broadly affects the expression of multiple DNA damage repair genes through regulating m^6^A modification via METTL3, and HDAC inhibitors can reverse chemotherapy resistance of TNBC by enhancing METTL3 lactylation and acting as targeted agents that block DNA damage repair.

Although, lactylation of METTL3 has been observed in TIM cells to facilitate the binding of METTL3 to RNA and augment immunosuppression.^[^ ^21^ ^]^ Nevertheless, the role of METTL3 in cancer development is context‐dependent, and lactylation may exhibit distinct biological functions in tumor cells and immune cells due to the intricate nature of the tumor microenvironment and metabolic milieu. Of note, the regulatory mechanism of METTL3 lactylation, particularly its negative regulation, as well as its biological function in tumor cells remain elusive. In our study, we have discovered that lactylation of METTL3 in TNBC cells does not impact its expression or nuclear–cytoplasmic distribution but can disrupt its interaction with WTAP. Since WTAP plays a pivotal role in localizing MTC to RNA, this weakened interaction directly leads to a reduction in global m^6^A modification levels. Additionally, we also observed a presence of lactylated METTL3 at basal levels in multiple breast cancer cells, indicating the existence of many independent METTL3 proteins outside the MTC. Intracellular interference with METTL3 expression does not have a more significant effect on m^6^A than METTL14 knockdown,^[^ ^58^ ^]^ and METTL14 has been identified as a tumor suppressor gene in colorectal cancer,^[^ ^59^, ^60^ ^]^ the contradictory role played by METTL3 and the underlying reasons for this discrepancy are unknown. The abundance of lactylated METTL3 may account for this disparity and inconsistency between METTL3 and METTL14, further suggesting that lactylated METTL3 might possess an undiscovered biological function independent of its complex and m^6^A modification function. This potential function could be crucial for the survival of breast cancer cells and other types of cancers.

UHRF1, ubiquitin‐like with PHD and RING finger domains 1, is indispensable for the mitotic inheritance of DNA methylation by targeting DNMT1 to replication forks.^[^ ^61^ ^]^ Moreover, UHRF1 also regulates de novo DNA methylation in vitro and in vivo by recruiting newborn DNMT (DNMT3A and DNMT3B) onto chromatin, facilitating these enzymes to establish new DNA methylation sites.^[^ ^62^ ^]^ Given that UHRF1 exhibits high expression levels in various tumors and exerts an oncogenic function, it plays a crucial role in the occurrence and progression of diverse tumor diseases. Therefore, from a clinical treatment perspective, UHRF1 holds promise as a potential therapeutic target. However, there are currently no specific inhibitors available for UHRF1 in cancer treatment. Our previous studies have reported that m^6^A regulates the expression of UHRF1.^[^ ^23^ ^]^ Herein, we further elucidate how does TNBC initiate m^6^A‐mediated UHRF1 regulation. In cisplatin‐resistant cells, HDAC2 delactylates METTL3 to upregulate m^6^A activity, thereby reactivating UHRF1 expression for DNA damage repair and conferring resistance against cell death.

HDAC inhibitors have received market approval due to their groundbreaking clinical efficacy in treating various subtypes of hematologic tumors, making them a successful example of targeted drugs for tumor epigenetic modification.^[^ ^63^ ^]^ In recent years, HDAC inhibitors have also demonstrated significant clinical effectiveness in diverse solid tumors.^[^ ^64^ ^]^ Tucidinostat, the first oral selective inhibitor targeting HDAC subtype, was approved in 2014 for the treatment of relapsed or refractory peripheral T‐cell lymphoma.^[^ ^38^ ^]^ While initially focused on blood disorders, Tucidinostat has shown promising potential for use across multiple tumor types. A phase II clinical study conducted in 2024 suggested that combining immune checkpoint inhibitors with antiangiogenesis drugs and the HDAC inhibitor Tucidinostat could be a promising therapeutic option for advanced colorectal cancer patients with MSS/PMMR status.^[^ ^65^ ^]^ Remarkable performance of Tucidinostat in breast cancer treatment was observed in 2023. The National Medical Products Administration of China has approved the combined use of Tucidinostat and aromatase inhibitors to enhance survival rates among hormone receptor‐positive/HER2‐negative breast cancer patients. However, whether or not Tucidinostat can participate in a wider variety of breast cancer treatment, and the specific molecular mechanism of Tucidinostat in the treatment of breast cancer has not been revealed, and how to better play the role of HDAC inhibitors in killing breast cancer tumors is imminent. This study has unveiled that Tucidinostat targeting HDAC2 directly enhances lactylation of METTL3 while concurrently suppressing genes associated with DNA damage repair. The combined treatment of Tucidinostat and cisplatin (even at low dose) exhibits remarkable efficacy in eliminating TNBC cells. These findings offer novel targets and insights for precision tumor therapy, underscoring their significant research value and clinical implications.

## 4 Conclusion

**Figure 8:**
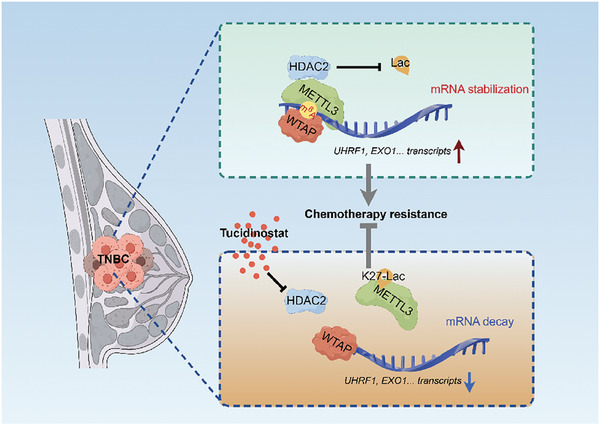
A schematic diagram of the underlying mechanism indicates that HDAC2‐mediated METTL3 delactylation promotes DNA damage repair and chemotherapy resistance in triple‐negative breast cancer.

In summary, our study employed molecular biological techniques pertaining to protein post‐translational modification, in conjunction with high‐throughput sequencing and bioinformatics analysis, to investigate the delactylation of METTL3 at its key lysine site through interaction with HDAC2. This process upregulates the global m^6^A modification and DNA damage repair pathway of TNBC cells. Targeted intervention of HDAC2 can enhance the sensitivity of TNBC to cisplatin chemotherapy resistance (**Figure** **8**). This study identifies the lactylation modification of METTL3 in breast tumors and its delactylase HDAC2. We elucidate the molecular mechanism by which HDAC2‐mediated METTL3 delactylation promotes TNBC resistance to cisplatin through upregulation of DNA damage repair pathway. The HDAC inhibitor Tucidinostat is proposed as a potential clinical approach for alleviating TNBC cisplatin resistance.

## 5 Experimental Section

### Study Approval

The mouse experiments conducted in this study were approved and carried out in accordance with the guidelines established by the Southern University of Science and Technology Laboratory Animal Center (SYXK2022‐0170).

### Cell Culture

HEK293T, MB231, SUM159, MB157, Hs578T, SKBR3, BT474, T47D, and MCF‐7 cell lines were initially obtained from ATCC. HEK293T cells were cultured in Dulbecco's modified Eagle medium (DMEM, Gibco) supplemented with 10% FBS (Gibco). MB231, Hs578T, and MCF‐7 cells were also maintained in DMEM supplemented with 10% FBS. T47D cells were cultured in RPMI1640 supplemented with 10% FBS and insulin. SUM159 cells were cultured in F12 medium supplemented with 10% FBS, insulin, and hydrocortisone. BT474 cells were maintained in Hybri‐Care medium supplemented with 10% FBS. SKBR3 cells were cultured in McCoy's 5A medium supplemented with 10% FBS. MB157 cells were cultured in DMEM‐F12 medium supplemented with 10% FBS. All the aforementioned cell lines were incubated at 37 °C under a saturated humidity atmosphere containing 95% air and 5% CO_2_ concentration. For HDACi treatment experiments, the respective cell lines were treated with the indicated time or doses of Tucidinostat. In RNA half‐life experiments, the indicated time involved exposing the cells to Actinomycin D at a concentration of 5 µg mL^−1^.

### Reagents and Antibodies

Tucidinostat (HY‐109015), TSA (HY‐15144), Oxamate (HY‐W013032A), and Cell Counting Kit‐8 (HY‐K0301) were purchased from MCE. Cisplatin (S1552) and 2‐deoxy‐d‐glucose (ST1024) were purchased from Beyotime. NAM (N0636), TRIzol (T9424), nuclease P1 (N8630), and alkaline phosphatase (P5931) were purchased from Sigma. Dynabeads purification kit (61006) and puromycin (A1113803) were purchased from Thermo Fisher Scientific. PrimeScript RT reagent Kit with gDNA Eraser (RR047A) was purchased from Takara. Taq Pro Universal SYBR qPCR Master Mix (Q712) was purchased from Vazyme. Protease inhibitor (04693132001) and RNase A (RNASEA‐RO) were purchased from Roche. DNase I (M0303) was purchased from NEB. Lactate assay kit (A019‐2‐1) was purchased from Jiancheng. Myc‐agarose (M20030) was purchased from Abmart. Anti‐METTL3 (15073‐1‐AP), anti‐METTL14 (26158‐1‐AP), anti‐WTAP (10200‐1‐AP and 60188‐1‐Ig), anti‐METTL16 (19924‐1‐AP), anti‐UHRF1 (21402‐1‐AP), anti‐EXO1 (16253‐1‐AP), anti‐Myc‐tag (16286‐1‐AP), anti‐GST‐tag (10000‐0‐AP), anti‐Actin (66009‐1‐Ig), anti‐GAPDH (60004‐1‐Ig), anti‐Tubulin (66240‐1‐AP), and anti‐Lamin B1 (66095‐1‐Ig) were obtained from Proteintech Group. Anti‐HDAC2 (ET1607‐78), anti‐FTO (ET1705‐89), anti‐ALKBH5 (ER65894), anti‐p300 (ER1908‐18), and anti‐γH2AX (ET1602‐2) were obtained from HUABIO. Anti‐ASF1B (A6511), anti‐MCM3 (A11475), and anti‐H3K18la (A21214) were obtained from ABclonal. Anti‐lactyl lysine (PTM‐1401) was obtained from PTM BIO. Anti‐Phospho‐METTL3 Ser43 (ABE2611) and anti‐Flag‐tag (F1804) were obtained from Sigma‐Aldrich. Anti‐m^6^A (202003, 2 µg µg^−1^ RNA for meRIP) was obtained from Synaptic Systems. Antiacetylated Lys (9441) was obtained from Cell Signaling Technology.

### Plasmid Constructs

The cDNAs encoding HDAC1, HDAC2, HDAC3, HDAC5, HDAC6, HDAC7, HDAC8, and HDAC10 were amplified and cloned into the pcDNA3.1 vector. To generate lentiviral expression constructs, the cDNAs encoding METTL3 and HDAC2 were amplified and cloned into the pLvx‐Flag vector. All mutant constructs of METTL3 or HDAC2 were generated using a PCR‐based site‐directed mutagenesis method with wild‐type METTL3 or HDAC2 constructs as templates. The plasmids (pLV‐C‐Flag‐vector) encoding UHRF1, EXO1, ASF1B, and MCM3 were gifts from Prof. Han You (Xiamen University, China).

### RNA Interference

The lentivirus‐based vector pLV‐H1‐EF1α (Biosettia) was utilized for the expression of shRNAs. The shRNA sequences targeting specific genes were as follows:

shMETTL3#1, 5′‐GGGCCCAAGTGCAAGAATTCT‐3′;

shMETTL3#2, 5′‐GCTGCACTTCAGACGAATT‐3′;

shHDAC2#1, 5′‐AGCTGTGAAGTTAAACCGACAA‐3′;

shHDAC2#2, 5′‐GCTGCTCAACTATGGTCTCTA‐3′;

shp300, 5′‐ATACTCAGCCGGAGGATATTT‐3′.

### Generation of DTP Cell Lines

The indicated TNBC cells reaching confluent were treated with following doses of cisplatin. For MB231 cells, 15 µm cisplatin was cultured for 72 h to obtain DTP cells, SUM159 cells were treated with 20 µm cisplatin for 60 h to obtain DTP cells, and MB157 cells were treated with 15 µm cisplatin for 60 h to obtain DTP cells. All cells were cultured with fresh medium containing the drug every 24 h.

### Generation of Cisplatin‐Resistant Cell Line

MB231 cells reaching ≈70% density in 10 cm dishes were treated with 0.2 µm cisplatin. And then, the concentration of cisplatin was gradually increased to IC50 for MB231 cells and maintained at the IC50 concentration for at least 4 passages. This continuous exposure was maintained for 6 months.

### Immunoblotting and Immunoprecipitation

Cells were lysed with RIPA buffer containing protease inhibitor cocktail and phenylmethylsulfonyl fluoride. For immunoprecipitation, cells were lysed in Triton buffer with protease inhibitor, followed by immunoprecipitation using Myc‐agarose beads, antilactyl lysine, or anti‐METTL3 antibody. Immune complexes were washed with Triton buffer, eluted in sodium dodecyl sulfate (SDS) sample buffer, and analyzed by immunoblotting. The formulas of RIPA and Triton buffer were conducted following previously described methods.^[^ ^11^ ^]^

### Quantitative RT‐PCR

Total RNA was extracted from samples using TRIzol reagents according to the manufacturer's instructions. cDNA was synthesized with RevertAid Reverse Transcriptase. Quantitative PCR was performed on the ABI7500 system. The qRT‐PCR primers are listed in Table S1 (Supporting Information).

### m^6^A Dot Blot

The total RNAs were denatured and transferred to an Amersham Hybond‐N+ membrane using a Bio‐Dot Apparatus. After UV cross‐linking, the membrane was stained with methylene blue, incubated with anti‐m^6^A antibody (#202003; Synaptic Systems) at 1:1000 dilution, and the m^6^A signal was quantified using Tanon Image software and normalized to methylene blue.

### RNA m^6^A Quantification by LC–MS/MS

The quantification of RNA m^6^A by LC–MS/MS was conducted following previously described methods.^[^ ^58^ ^]^ Briefly, polyadenylated RNAs were extracted using the Dynabeads mRNA purification kit (Thermo Fisher Scientific) and quantified with Qubit. 200 ng of mRNA was digested with nuclease P1 and alkaline phosphatase at 37 °C for 2 h, respectively. The solution was centrifuged and injected into LC–MS/MS. The m^6^A to A ratio was calculated based on these concentrations.

### Lactate Concentration Measure

Intracellular lactate level was measured using lactate assay kit (Jiancheng, A019‐2‐1) according to manufacturer's protocol.

### Subcellular Fractionation

Cells were lysed in hypotonic buffer on ice for 15 min. NP‐40 was added to a final concentration of 0.25% and incubated for 5 min. Samples were centrifuged at 425 × *g* for 5 min at 4 °C to collect the cytoplasmic fraction as supernatant. The nuclear pellet was washed with hypotonic buffer and resuspended in hypertonic buffer, then incubated on ice for 30 min. After centrifugation at 20 000 × *g* for 5 min, the nuclear fractions were collected as supernatants. The formulas of hypotonic and hypertonic buffer were conducted following previously described methods.^[^ ^11^ ^]^

### Immunofluorescence Staining

The protocol was conducted following previously described methods.^[^ ^11^ ^]^ Briefly, cells on glass coverslips were washed with phosphate‐buffered saline (PBS), fixed with 4% paraformaldehyde, and permeabilized with 0.5% Triton X‐100. Cells were then blocked with 1% bovine serum albumin and incubated overnight at 4 °C with the primary antibody. Next, cells were incubated with DAPI and secondary antibodies (Alexa Fluor 594 or 488). Images were captured using a Zeiss LSM980 confocal microscope with ZEN2010 software.

### In Vitro Delactylation Assays

The reaction followed previously described methods.^[^ ^20^ ^]^ GST fusion constructs were expressed in BL21 *Escherichia coli*, and crude lysates were prepared by sonication in cold NETN buffer with protease inhibitors. Glutathione–sepharose beads purified GST–HDAC2. For delactylation, Myc‐tagged METTL3 from transfected HEK293T cells was immunoprecipitated using Myc‐antibody‐conjugated beads. The beads were washed 3 times with lysis buffer and twice with 25 mm Tris‐HCl (pH 7.5). Myc‐tagged lactylated METTL3 was eluted with Myc peptides in 25 mm Tris‐HCl (pH 7.5) buffer containing 150 mm NaCl and protease inhibitors, then incubated with GST–HDAC2 in Tris buffer for 1 h at 37 °C. The reaction was stopped with SDS sample buffer, and samples were analyzed by SDS–polyacrylamide gel electrophoresis and western blotting.

### Real‐Time Monitoring Cell Viability

The xCELLigence RTCA DP instrument (ACEA Biosciences) was placed in a humidified incubator at 37 °C with 5% CO_2_ for real‐time monitoring of live cells. To measure the background signal from cell‐free medium, 50 µL of culture medium was added to each well of 16‐well plates (ACEA Biosciences, 00300600880). Cells were seeded and cultured under specified treatments with a range of 500–2000 cells per well. Each condition was performed in triplicate, and a programmed signal detection schedule monitored the cells every 4 h for a period ranging from 36 to 180 h.

### Clonogenic Survival Assay

The clonogenic survival assay was conducted according to previously established protocols.^[^ ^23^ ^]^ 2000–5000 cells were seeded in 6‐well plates and allowed to adhere for 24 h. Cells were then treated with specified drugs. The medium was refreshed every three days, maintaining drug concentrations for 14 days. Colonies were fixed with methanol, stained with crystal violet, and scanned digitally. Results were normalized to OD measurements at 570 nm.

### Cell Proliferation Assay

Cells (1000–3000 cells per well) were seeded onto 96‐well plates with 100 µL of complete medium. Cell proliferation was assessed using the Cell Counting Kit‐8 at the designated time points. For drug sensitivity assays, cells (1000–3000 cells per well) were plated on 96‐well plates overnight and subsequently treated with specified concentrations of compounds. After 72 h of drug exposure, cytotoxicity was evaluated using the CCK‐8 kit.

### Migration/Invasion Assays

The protocol was conducted following previously described methods.^[^ ^11^ ^]^ For the migration assay, an 8 µm Transwell (3422, Corning) was used. Cells were seeded in the upper chamber with 0.2% serum medium, while the lower chamber contained 10% FBS medium as a chemoattractant. For the invasion assay, an 8 µm BD Matrigel Invasion Chamber was used. Cells were incubated for 6 h for migration and 12 h for invasion. After incubation, cells on the upper side were removed, and those adhering to the underside were fixed with methanol and stained with crystal violet.

### MeRIP–qPCR

The meRIP–qPCR experiment was conducted following the previously described protocol.^[^ ^66^ ^]^ Briefly, poly(A)+ RNAs were purified with the Dynabeads mRNA kit, fragmented, and immunoprecipitated with an anti‐m^6^A antibody (Synaptic Systems) in IP buffer for 2 h at 4 °C. The m^6^A–IP mixture was incubated with Dynabeads protein A (Thermo Fisher Scientific) for another 2 h at 4 °C. Bound RNA was eluted with *N* ^6^‐methyladenosine (Berry & Associates) and purified using a Zymo Research RNA cleanup kit. Fragmented mRNA was used for meRIP–qPCR analysis. Primer sequences can be found in Table S2 (Supporting Information).

### Computational Analysis of RNA‐Seq Data

The sequencing reads were aligned to the human reference genome (hg38) using the STAR aligner. Cufflinks calculated RefSeq gene expression, with the options ‐M and ‐u utilized for masking reads aligned to repetitive regions and correcting multiple aligned reads using the “rescue method.” Genes with FPKM > 0.5 in specified samples were analyzed. Differentially expressed genes were identified by a fold change > 1.5. The heatmap was generated with deepTools, and significance was determined through Student's *t*‐test.

### Animal Studies

The mice were housed in specific pathogen‐free conditions with a 12 h light/12 h dark cycle at a temperature of 22 ± 2 °C and a humidity level of 50 ± 5%. They were provided with a standard mouse chow diet at the Southern University of Science and the Technology University Laboratory Animal Center. For all experiments, female BALB/c nude mice aged six weeks were utilized. In MB231 xenograft experiments, the indicated MB231 or MB231‐R cells (2 × 10^6^) suspended in a solution of PBS and Matrigel matrix (1:1) were subcutaneously injected into the mice using a volume of 100 µL (mice *n* = 5 per group). Once tumors formed, the mice were randomly assigned to receive either vehicle, cisplatin (2 mg kg^−1^ once per week), Tucidinostat (15 mg kg^−1^), or both agents in combination. The drugs were administered, and tumor volume was measured every other day for the specified duration using the formula: (width^2^ × length)/2. When tumors reached a certain size, the mice were euthanized for tissue collection, and tumor tissues were excised and photographed.

### Statistics and Reproducibility

The data were presented as mean ± standard deviation (SD), as indicated, from three independent experiments or biological replicates. Statistical significance was determined using two‐tailed Student's *t*‐test with Prism 8.0.2 software (GraphPad Software), unless otherwise specified. *p* value less than 0.05 was considered statistically significant.

## Conflict of Interest

The authors declare no conflict of interest.

## Author Contributions

X.H., Y.L., and J.L. contributed equally to this work. X.H. and Y.L. conceived and designed the study. X.H., Y.L., and S.C. performed most of the experiments. Z.X. helped to order the reagents. X.H., Y.L, and J.L. analyzed the data. X.H., Y.L., and X.Y. wrote the paper. J.D., G.C., J.S., and Q.M. revised the paper. All authors read and approved the final paper.

## Supporting information



Supporting Information

## Data Availability

The data that support the findings of this study are available from the corresponding author upon reasonable request.
