# Supplementary material for: HDAC2‐Mediated METTL3 Delactylation Promotes DNA Damage Repair and Chemotherapy Resistance in Triple‐Negative Breast Cancer
Source: Adv Sci (Weinh). 2025 Feb 14;12(14):2413121. doi: 10.1002/advs.202413121 (PMC11984901; doi:10.1002/advs.202413121)
Supplement: Supplementary file 1 — Supporting Information [file ADVS-12-2413121-s001.docx]

**Supporting Information**

**HDAC2-Mediated METTL3 Delactylation Promotes DNA Damage Repair and Chemotherapy Resistance in Triple-Negative Breast Cancer**

**Authors**

Xiaoniu He^1,7^, Yuanpei Li^2,7^, Jian Li^3,7^, Yu Li^4^, Sijie Chen^4^, Xia Yan^1^, Zhangrong Xie^4^, Jiangfeng Du^5#^, Guoan Chen^4#^, Jianbo Song^1#^and Qi Mei^6,8#^

**Content:**

**Supplementary Figure legends**

**Figure S1.** Lactate metabolic pathway governs the METTL3 basal lactylation in TNBC cells

**Figure S2.** K27 site of METTL3 undergoes direct delactylation mediated by HDAC2

**Figure S3.** The delactylation of METTL3 by HDAC2 regulates its interaction with WTAP, but does not affect its expression

**Figure S4.** High expressed HDAC2 is indispensable for the growth of TNBC under cisplatin

**Figure S5.** Delactylation of METTL3 by HDAC2 up-regulates DNA damage repair pathway in TNBC under cisplatin

**Figure S6.** Function rescue experiments for DNA damage repair related targets

**Figure S7.** Cisplatin sensitivity of TNBC is enhanced by Tucidinostat through inhibition of DNA damage repair pathways

**Figure S8.** Combination therapy of Tucidinostat and cisplatin for cisplatin-resistant breast cancer cells

**Table S1.** Primers for qRT-PCR

**Table S2.** Primers for meRIP qRT-PCR

**Supplementary Figure legends**

**Figure S1.** Lactate metabolic pathway governs the METTL3 basal lactylation in TNBC cells. A) Histogram shows the median mRNA expression profile of *METTL3* across the indicated tumor samples (red) and paired tissues (blue) using TCGA database. B) Lysates of parental and cisplatin tolerant MB157, SUM159, and MB231 cells were subjected to IP and IB analysis. C) Schematic representation of metabolic pathway of glycolysis and lactate production. D) IB of WCL (whole cell lysate) and proteins immunoprecipitated with anti-Myc beads from MB231 cells expressing Myc-METTL3 treated with Oxamate (20 mM) or 2-DG (10 mM) for indicated time period. E) IB of WCL (whole cell lysate) and proteins immunoprecipitated with anti-Myc beads from MB231 cells expressing Myc-METTL3 treated with lactate (25 mM) or sodium lactate (NaLac, 25 mM) for 24 h. Data are representative of three independent experiments (B,D,E).

**Figure S2.** K27 site of METTL3 undergoes direct delactylation mediated by HDAC2. A) Lysates of MB231 cells were subjected to IP and IB analysis. B) IB analysis of the indicated proteins in parental and cisplatin-tolerant MB231, SUM159 and MB157 cells. C) Intracellular lactate measurement in parental and cisplatin-tolerant MB231, SUM159 and MB157 cells. D) Co-IP of HDAC2 with METTL3 in MB157 or Hs578T cells. The immunoprecipitated materials by the indicated antibodies were analyzed by western blotting. E) IP and IB analysis of SUM159 cells infected with the indicated lentiviruses. F) IP and IB analysis of SUM159 cells treated with HDAC2 inhibitor Tucidinostat (1 μM) for 72 h. G) Sequences alignment of the residues flanking Lys 27 across different species. Arrowhead points to the Lys 27 residue corresponding to human METTL3. H) Incubation of the indicated recombinant HDAC2 proteins with lactylated Myc-METTL3 proteins, followed by IB analysis of the lactylation level of METTL3 with anti-Kla. Data are representative of three independent experiments. Mean ± SD, statistical analysis was performed using two-tailed Student’s *t*-test (C). ns, no significance.

**Figure S3.** The delactylation of METTL3 by HDAC2 regulates its interaction with WTAP, but does not affect its expression. A,B) Lysates of MB231 cells infected with the indicated lentiviruses combined 1 μM cisplatin treatment for 24 h were subjected to IB analysis. C) Dot blot of m^6^A in total RNA from indicated cells combined cisplatin treatment (1 μM) for 24 h. MB staining served as RNA loading control in dot blot analysis. D) Lysates of METTL3 reconstituting MB231 cells infected with the indicated lentiviruses combined 1 μM cisplatin treatment for 24 h were subjected to IB analysis. E) Relative mRNA levels of the indicated mRNAs in MB231 cells infected with the indicated lentiviruses combined 1 μM cisplatin treatment for 24 h were analyzed by qRT-PCR. F) Relative mRNA levels of the indicated mRNAs in SUM159 cells infected with the indicated lentiviruses combined 1 μM cisplatin treatment for 24 h were analyzed by qRT-PCR. G) IB analysis of cytoplasmic (Cyto) and nuclear (NE) fractions from the MB231 and SUM159 cells infected with the indicated lentiviruses combined 1 μM cisplatin treatment for 24 h. H) IP and IB analysis of SUM159 cells infected with the indicated lentiviruses combined 1 μM cisplatin treatment for 24 h. All data are representative of three independent experiments. Mean ± SD, statistical analysis was performed using two-tailed Student’s *t*-test (E,F). ***, *P* < 0.001. ns, no significance.

**Figure S4.** High expressed HDAC2 is indispensable for the growth of TNBC under cisplatin. A) Histogram shows the median mRNA expression profile of *HDAC2* across the indicated tumor samples (orange) and paired tissues (green) using TCGA database. The cancer types with *HDAC2* expression levels greater are highlighted in red. B) *HDAC2* expression profiled in BRCA by GEPIA. Tumor, red box; normal tissues, grey box. C) Kaplan-Meier analysis of overall survival in breast cancers according to high (red curve) or low (blue curve) *HDAC2* mRNA expression levels. D,E) Cell proliferation assays for MB231 and SUM159 cells infected with indicated lentiviruses combined 1 μM cisplatin treatment. F) IB analysis shows HDAC2 wild-type and H142A reconstituting cell lines construction. G,H) Cell proliferation assays for MB231 and SUM159 cells infected with indicated lentiviruses combined 1 μM cisplatin treatment. Data are representative of three independent experiments (D,E,F,G,H). Mean ± SD, statistical analysis was performed using two-tailed Student’s *t*-test (B,D,E,G,H) or log rank test (C). *, *P* < 0.05. ***, *P* < 0.001.

**Figure S5.** Delactylation of METTL3 by HDAC2 up-regulates DNA damage repair pathway in TNBC under cisplatin. A) Gene ontology enrichment analysis of differential expressed transcripts between HDAC2-deficient and Control MB231 cells. B) Gene ontology functional enrichment analysis of overlapped genes in Figure 6D. C) Genomic visualization of the meRIP-seq normalized signal in METTL3-dificient MB231 cells for the METTL3-dependent m^6^A substrates *ASF1B*, *MCM3*. Blue, meRIP; gray, input. D,E) m^6^A levels in *UHRF1*, *EXO1*, *ASF1B* and *MCM3* transcripts determined by gene-specific m^6^A RIP-qPCR assays in METTL3 or HDAC2 knockdown MB231 (D) and SUM159 (E) cells. F,G) Relative mRNA levels of the indicated mRNAs in MB231 (F) and SUM159 (G) cells infected with the indicated lentiviruses combined 1 μM cisplatin treatment for 24 h were analyzed by qRT-PCR. H) Reducing *UHRF1*, *EXO1*, *ASF1B* and *MCM3* mRNA half-life by silencing METTL3 or HDAC2 in SUM159 cells. I,J) Relative mRNA levels of the indicated transcripts in METTL3 reconstituting MB231 (I) and SUM159 (J) cells infected with the indicated lentiviruses combined 1 μM cisplatin treatment for 24 h were analyzed by qRT-PCR. K) Representative images of γH2AX foci in METTL3 reconstituting SUM159 infected with indicated lentiviruses upon 1 μM cisplatin treatment for 24 h. Scale bars, 10 μm. L) Spearman’s correlation coefficient analysis the indicated genes in breast cancers. Data are representative of three independent experiments (D,E,F,G,H,I,J,K). Mean ± SD, statistical analysis was performed using two-tailed Student’s *t*-test (D,E,F,G,I,J). **, *P* < 0.01. ***, *P* < 0.001. ns, no significance.

**Figure S6.** Function rescue experiments for DNA damage repair related targets. A,D,G,J) IB analysis of the indicated proteins in MB231 cells infected with the indicated lentiviruses combined cisplatin treatment (1 μM) for 24 h. B,E,H,K) Representative immunofluorescence (IF) for γ-H2AX (green) and DAPI (blue) in MB231 cells infected with the indicated lentiviruses combined cisplatin treatment (1 μM) for 24 h. Scale bars, 10 μm. C,F,I,L) Clonogenic assay (left) and quantification (right) of MB231 cells infected with the indicated lentiviruses combined indicated cisplatin treatment and Tucidinostat (0.5 μM) treatment for 14 days. All data are representative of three independent experiments. Mean ± SD, statistical analysis was performed using two-tailed Student’s *t*-test (C,F,I,L). *, *P* < 0.05. **, *P* < 0.01. ***, *P* < 0.001.

**Figure S7.** Cisplatin sensitivity of TNBC is enhanced by Tucidinostat through inhibition of DNA damage repair pathways. A) Cisplatin dose-response curves for SUM159 cells infected with HDAC2 shRNA or treated with 0.5 μM Tucidinostat as detected by CCK8 assay. B) Cisplatin dose-response for clonogenic assay and quantification of SUM159 subjected to HDAC2 shRNA or 0.5 μM Tucidinostat administration for 14 days. C) Representative images of γH2AX foci in SUM159 subjected to 0.5 μM Tucidinostat administration or/and 1 μM cisplatin treatment for 24 h. Scale bars, 10 μm. D,E) Cisplatin dose-response for clonogenic assay and quantification of MB231 and SUM159 subjected to HDAC2 shRNA or 0.5 μM Tucidinostat administration for 14 days. F) IB analysis of the indicated proteins in xenografted tumors of Figure 7E. Data are representative of three independent experiments (A,B,C,D,E,F). Mean ± SD, statistical analysis was performed using two-tailed Student’s *t*-test (B,D,E). ***, *P* < 0.001. ns, no significance.

**Figure S8.** Combination therapy of Tucidinostat and cisplatin for cisplatin-resistant breast cancer cells. A) Cell viability analysis of parental and cisplatin resistant MB231 cells using CCK8 assay after treatment with cisplatin. B) Lysates of parental and cisplatin resistant MB231 cells were subjected to IP and IB analysis. C) Dot blot of m^6^A in total RNA from parental and MB231-R cells. D,E) Clonogenic assay (D) and quantification (E) of MB231-R cells treated with cisplatin (indicated doses) and Tucidinostat (0.5 μM) for 14 days. F) Experimental scheme of drug administration of xenografted mouse model of cisplatin-resistant MB231 cells. G–I) MB231-R cells were subcutaneously injected into BALB/C nude mice and treated as indicated in (F). Tumor images were shown in (G); tumor volumes were calculated as described in (H); tumor weights were measured in (I). Data are representative of three independent experiments (A,B,C,D). G,H,I, *n* = 5. Mean ± SD, statistical analysis was performed using two-tailed Student’s *t*-test (E,H,I). *, *P* < 0.05. ***, *P* < 0.001.


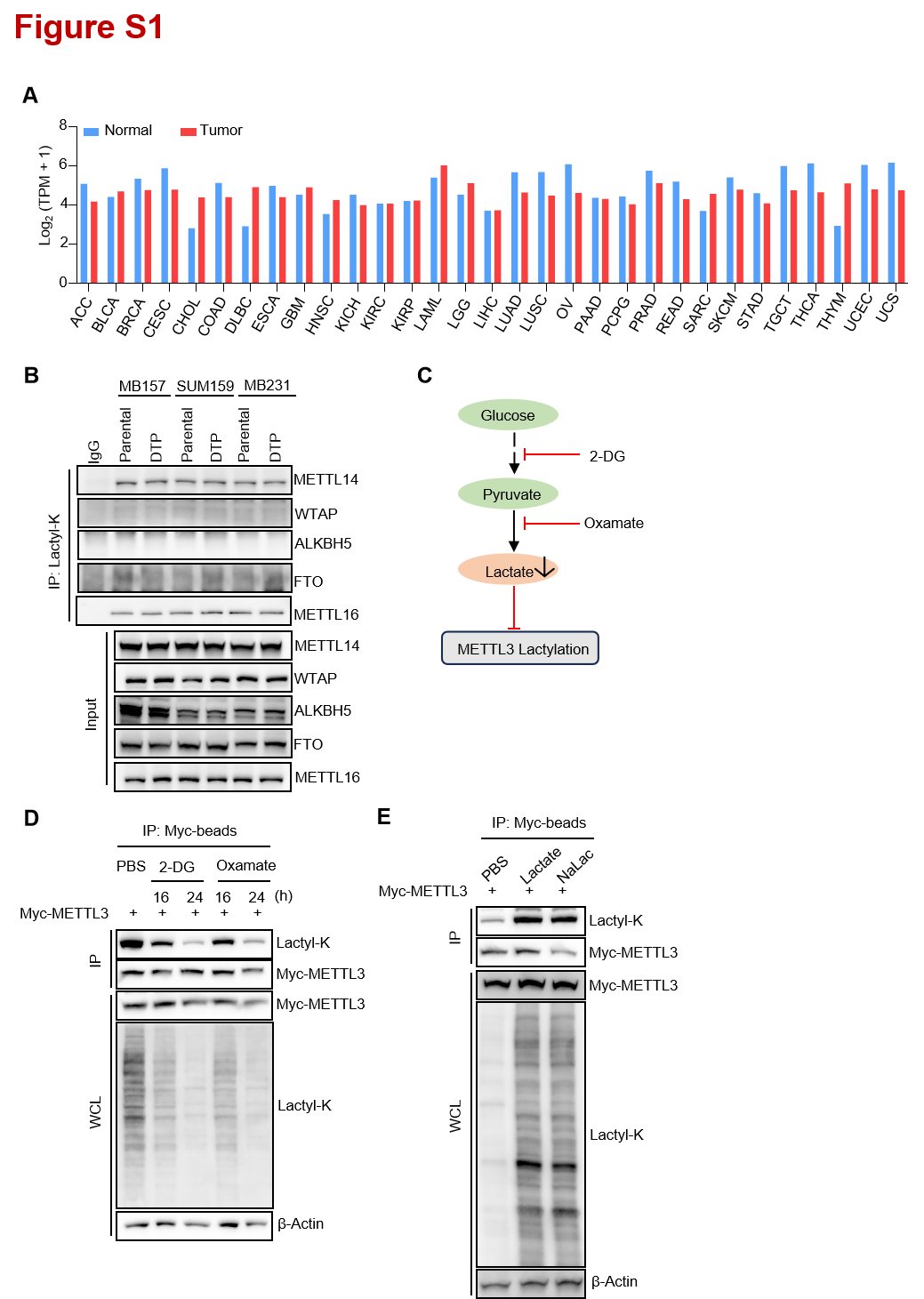


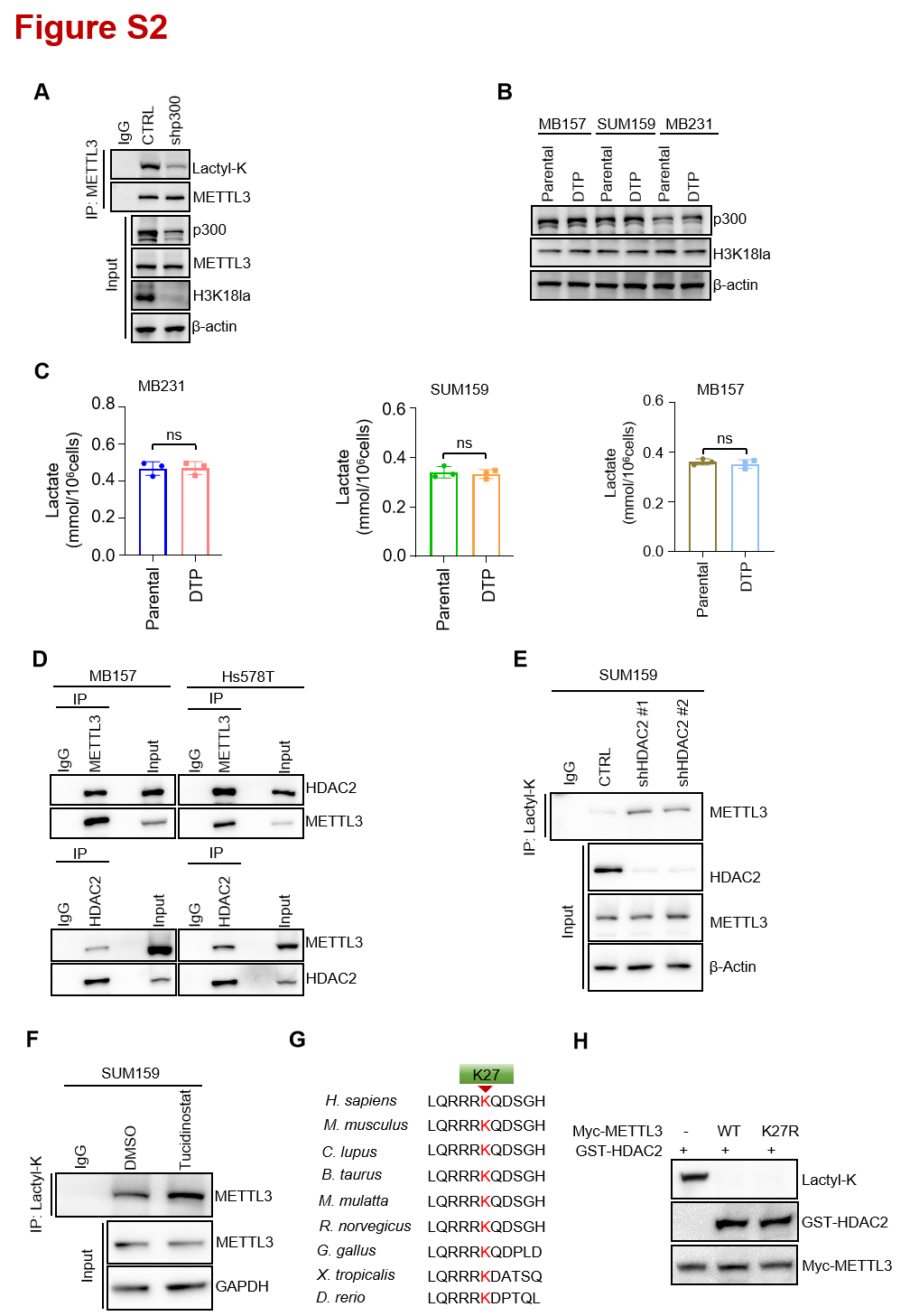


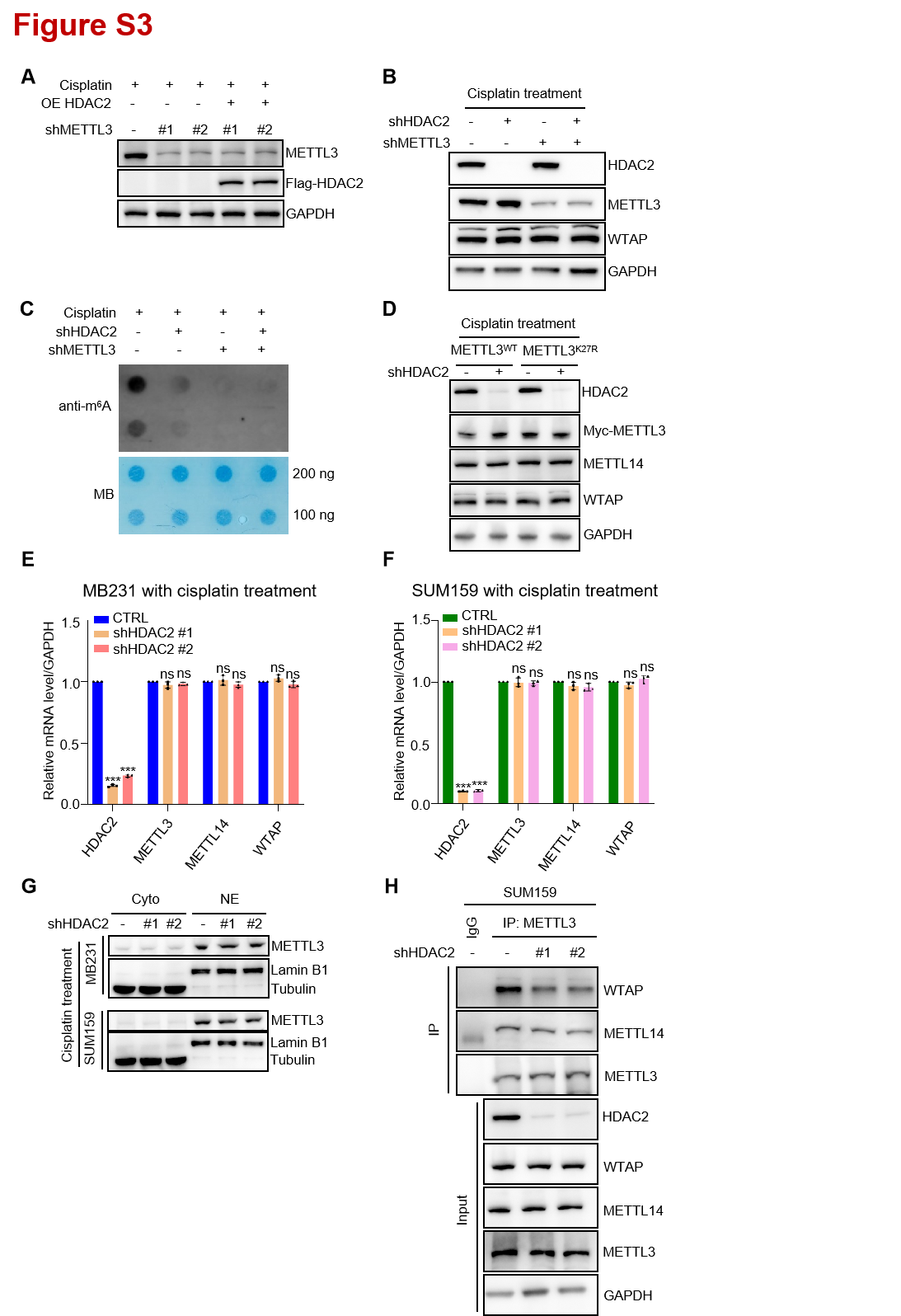


**
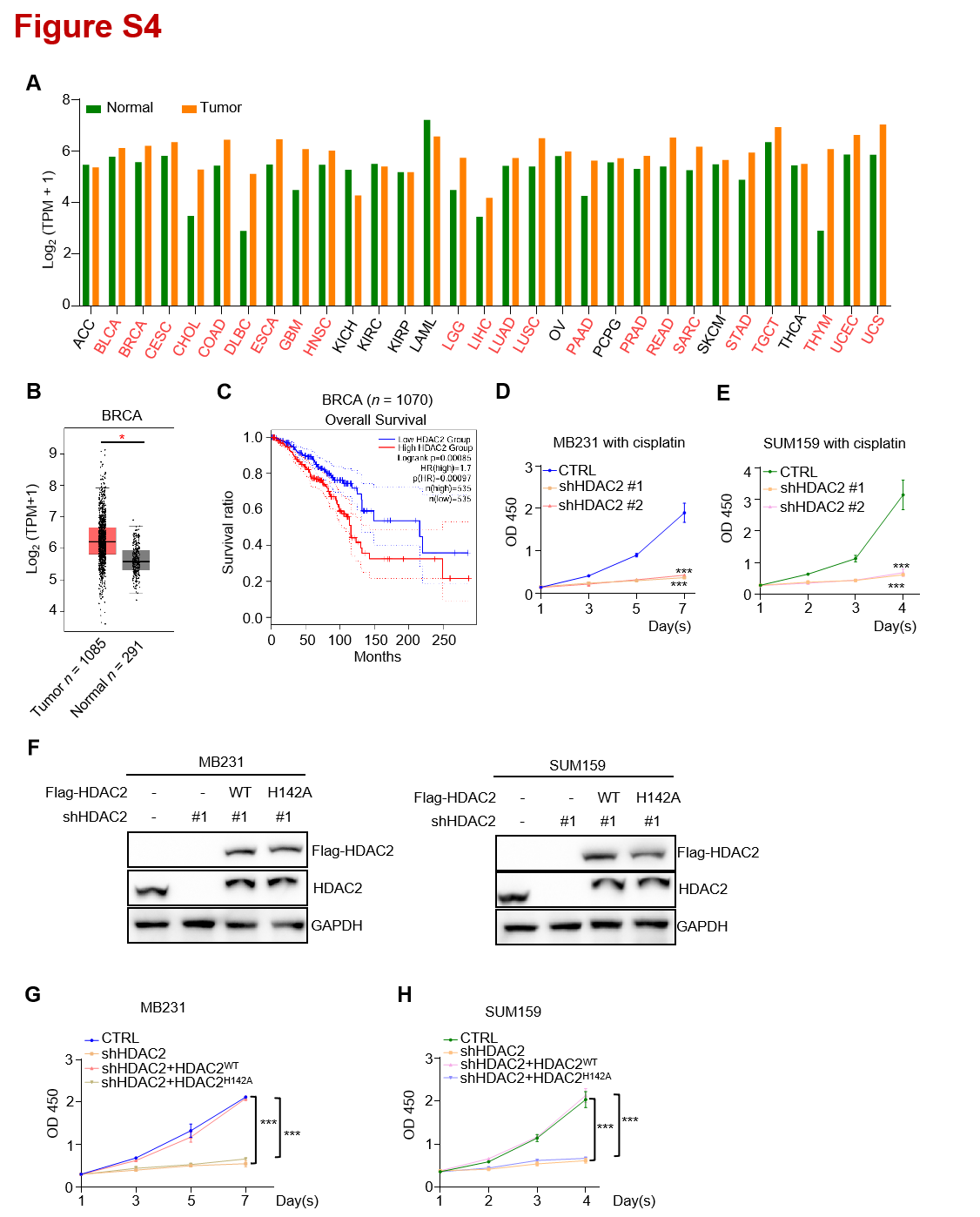
**

**
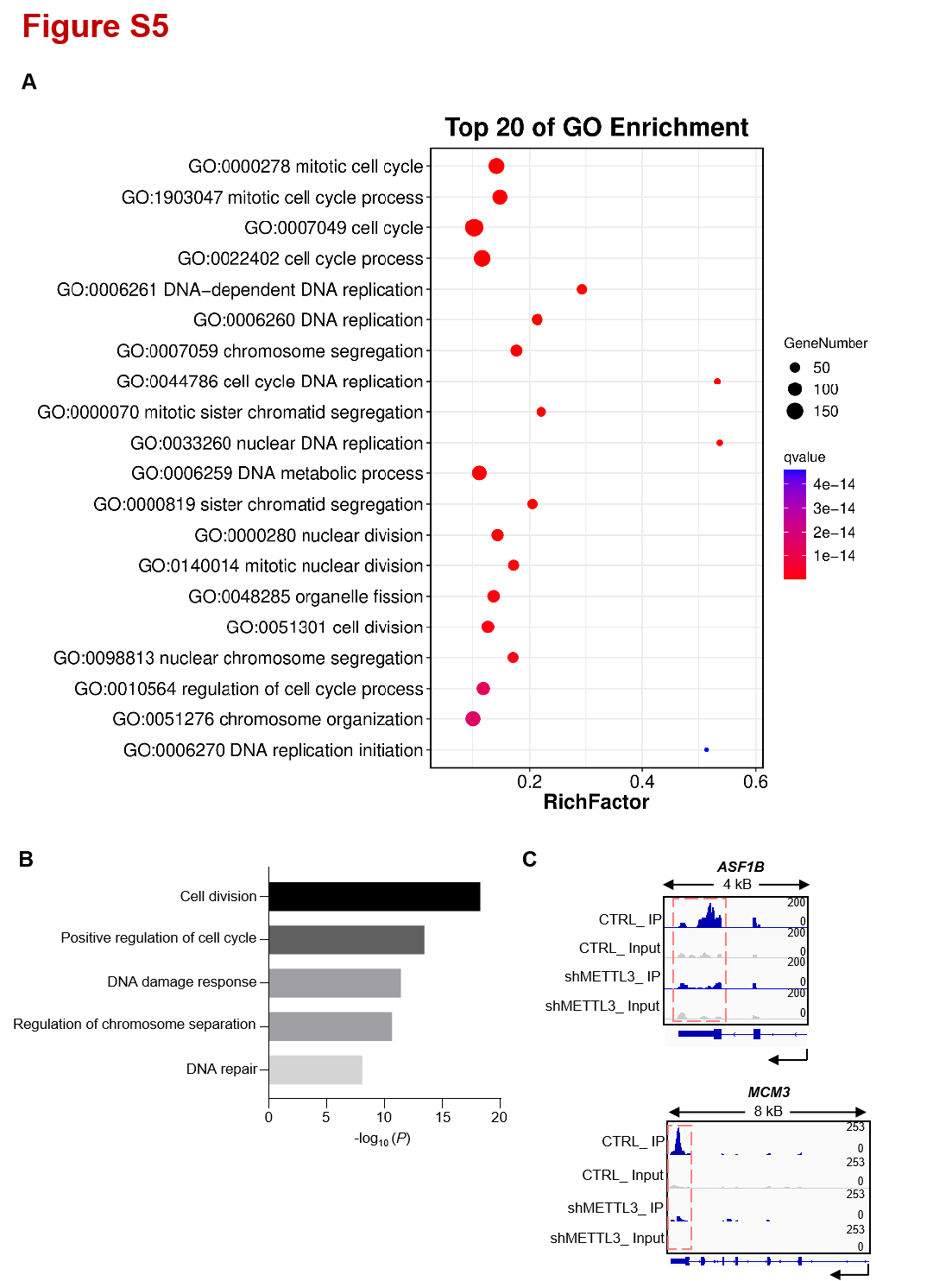

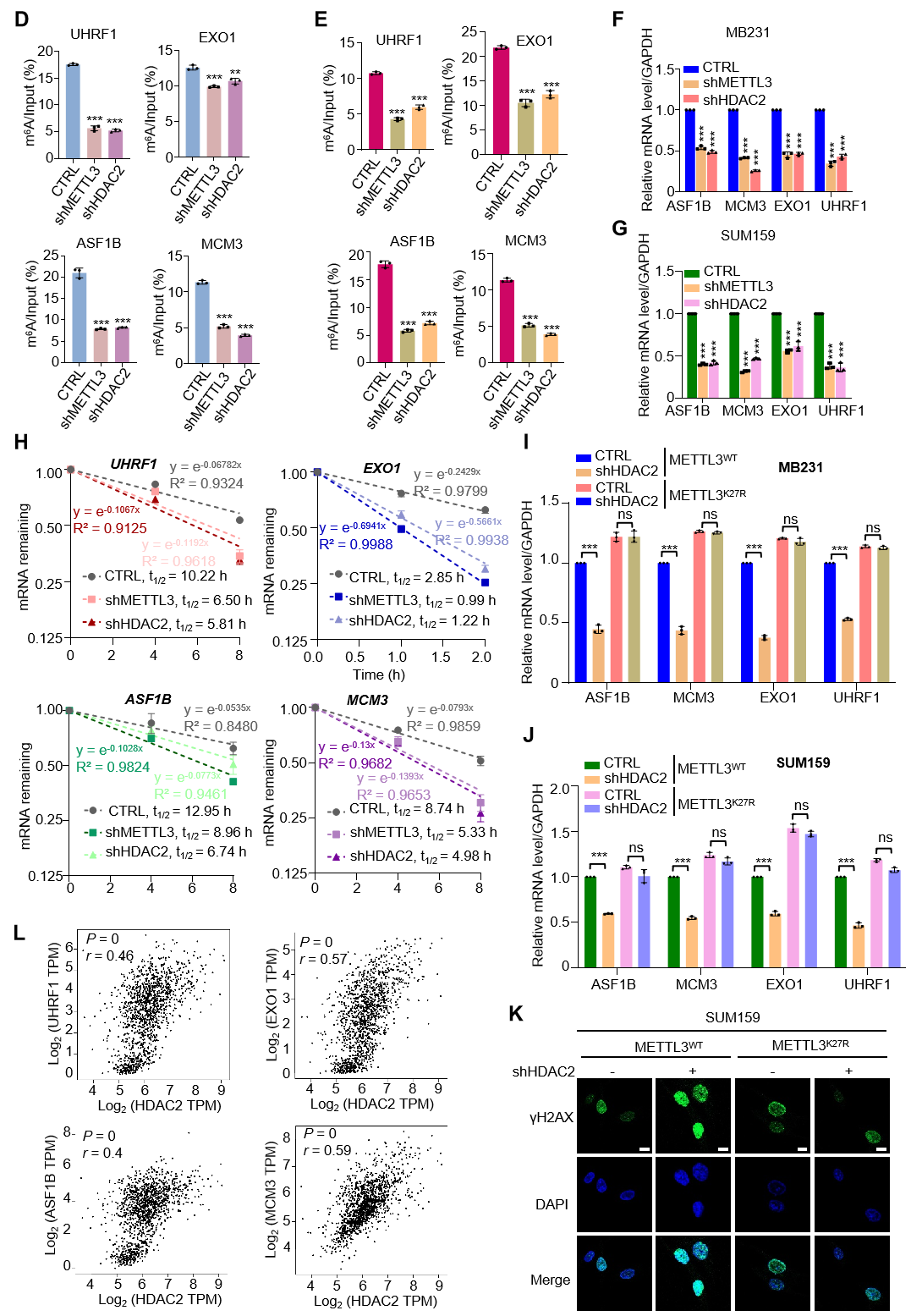
**

**
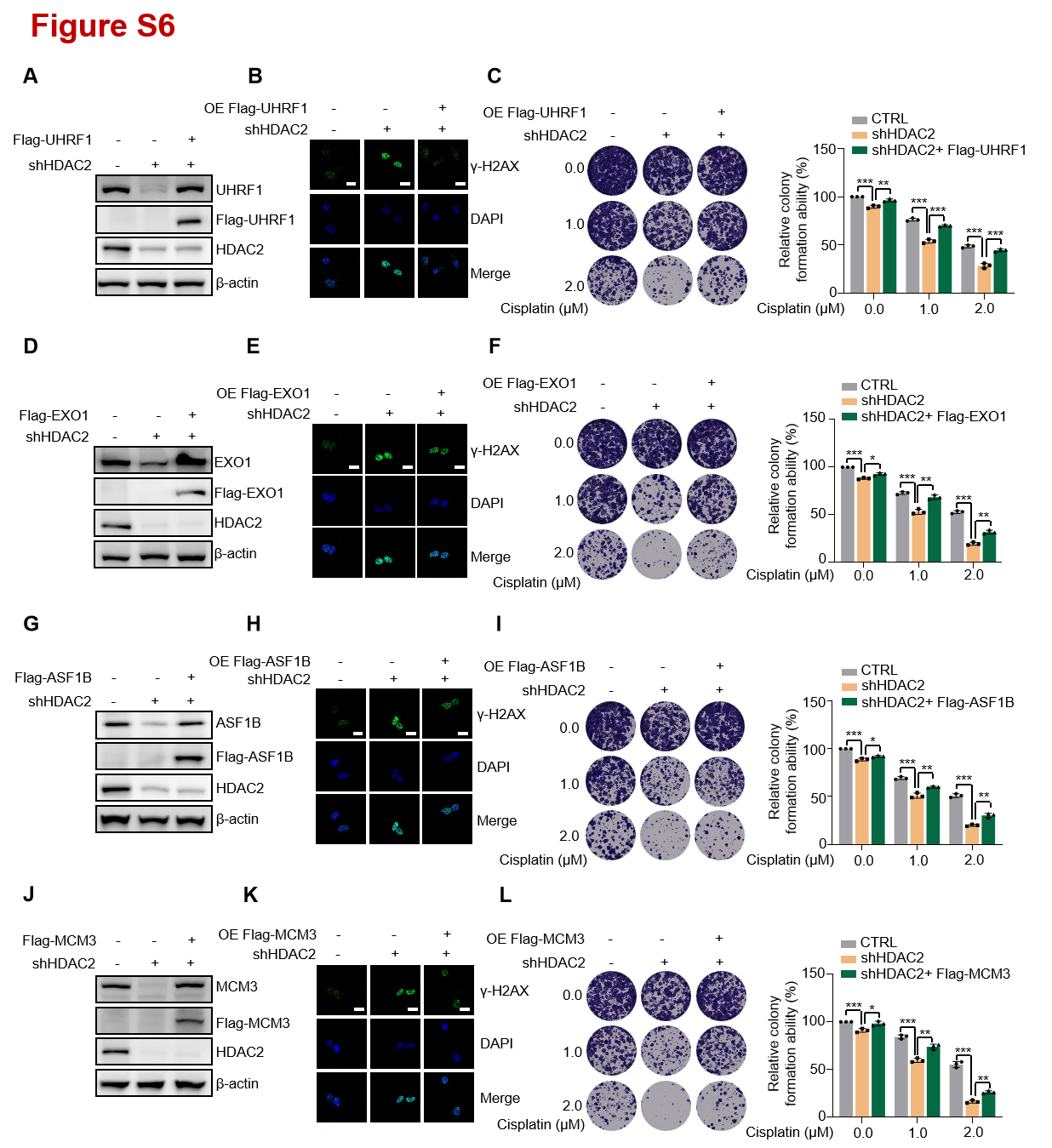
**

**
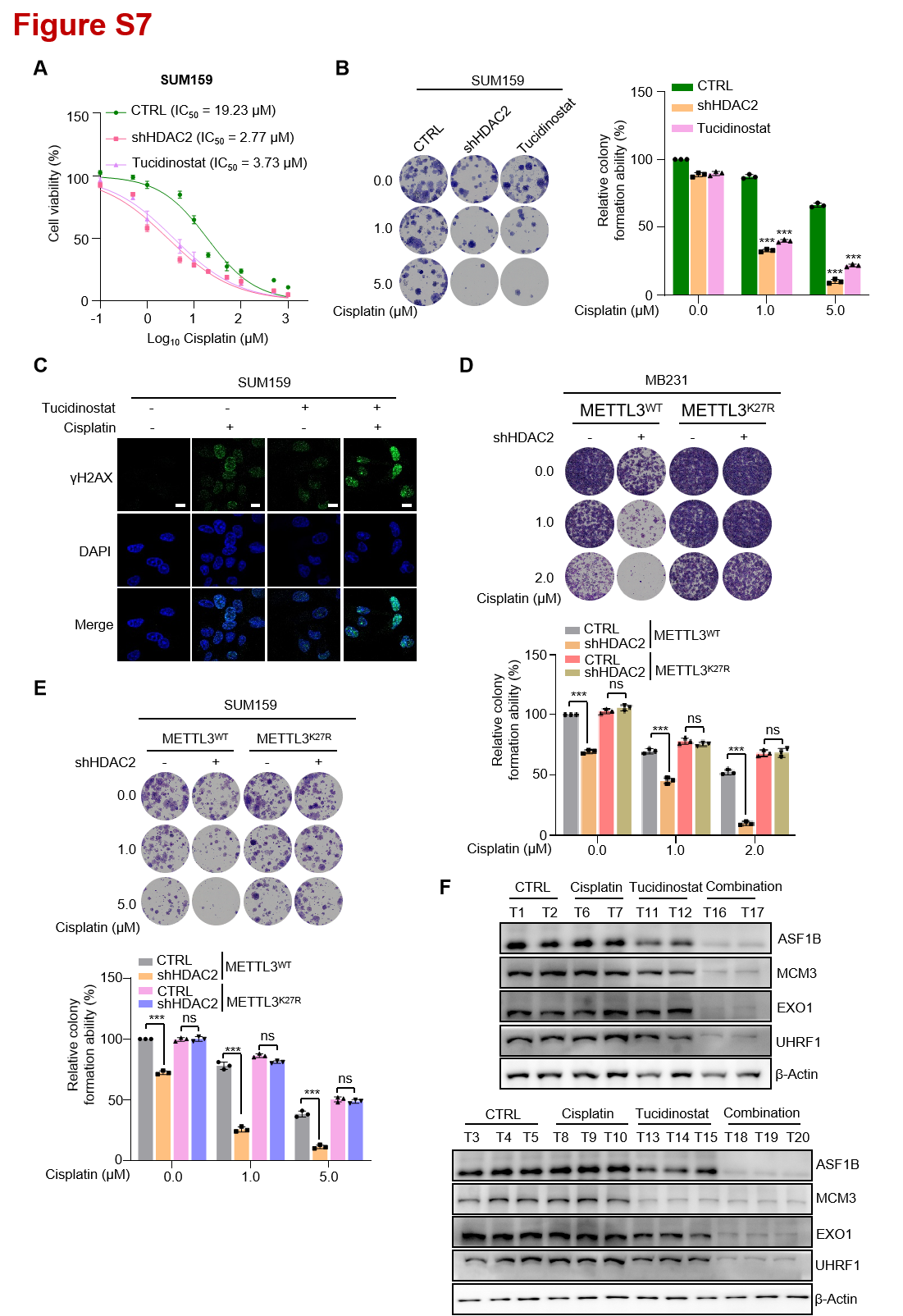
**

**
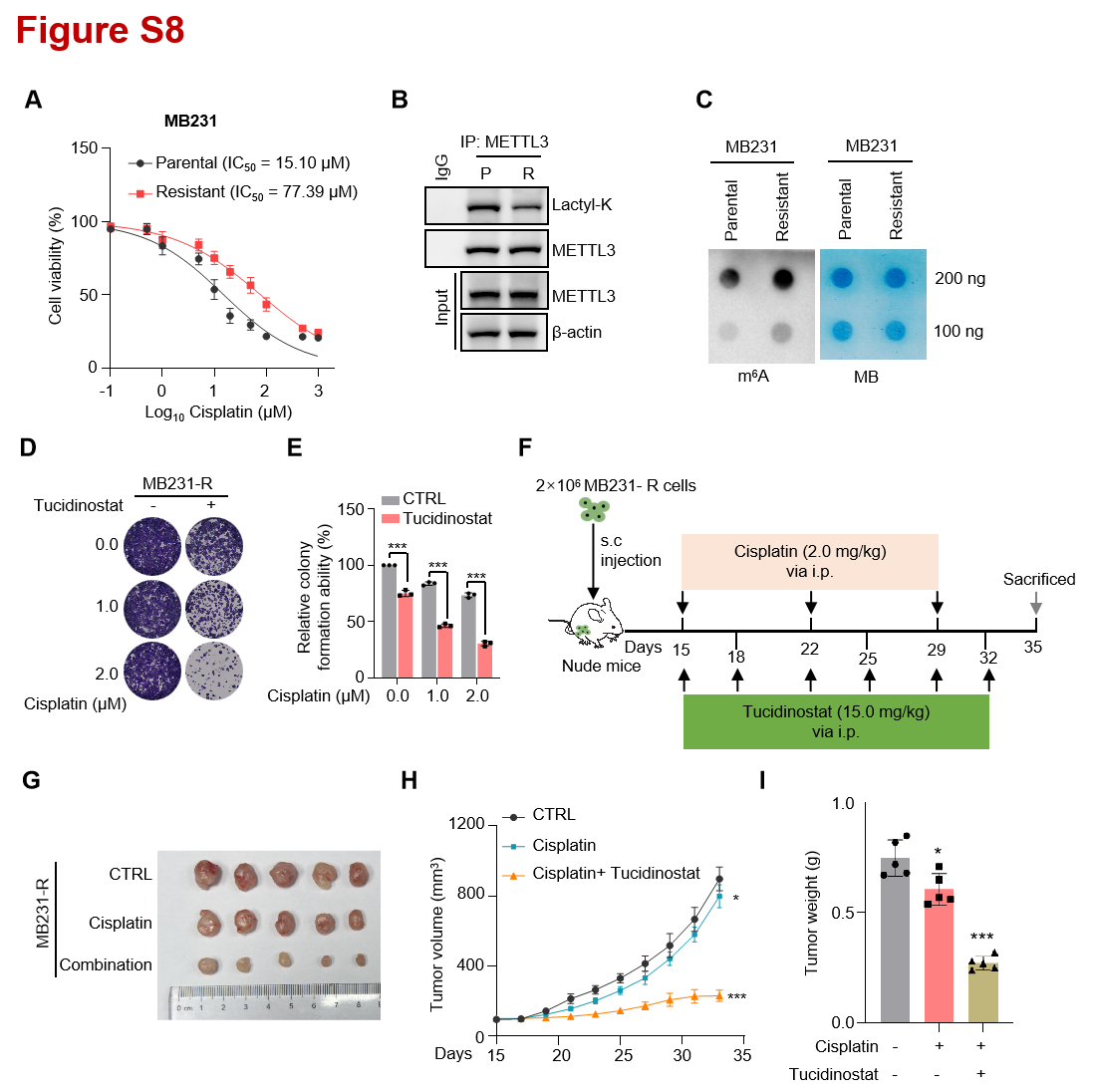
**

**Table S1.** Primers for qRT-PCR

| Target Forward primer (5’—3’) Reverse primer (5’—3’) |
| --- |
| *METTL3* ATCCCCAAGGCTTCAACCAG GCGAGTGCCAGGAGATAGTC  *METTL14* GGGGTTGGACCTTGGAAGAG TTGGTCCAACTGTGAGCCAG  *WTAP* ACAAGCTTTGGAGGGCAAGT GCGGGAGGAGCTACCATTAC  *HDAC2* CTGCTACTACTACGACGGTGA GTCATTTCTTCGGCAGTGGC  *ASF1B* GTTTGTCTTTCAGGCCGACG ACGTAGTAGCCCACTCGGAT  *MCM3* TGGAGTCATCCTGGGAACCT TTGTTCAGAAGCCTCGTCGT  *EXO1* GAACAAGCCGGGGTTACAGA CACGGCCACATTCAGGTTTG  *UHRF1* CACAACGTGTGCAAGGACT CACAACGTGTGCAAGGACTG  *GAPDH* CCTGACCTGCCGTCTAGAAA CCCTGTTGCTGTAGCCAAAT |

**Table S2.** Primers for meRIP qRT-PCR

| Target Forward primer (5’—3’) Reverse primer (5’—3’) |
| --- |
| *ASF1B* TCCCAGTTGATATGGAAGCGG GGCGTACCTACAAGGGTTGAC  *MCM3* GAGGCGATTCATGCCGATTG CTGTCTGGGGCAGGTTGAAG  *EXO1* CCACCCACTTTGGGAACACT GCAATGCTGTGCTTGGAGAG  *UHRF1* TCCTATTTGCAAACTGCCTTTGG TGCCTCATGTTTCTCCTGGGATT |
